# The underlying mechanism of scorpion venom peptide BmK AS in reducing epilepsy seizures: mediated through dual modulation of Nav1.6 and the inflammasome pathway

**DOI:** 10.3389/fphar.2026.1747856

**Published:** 2026-04-15

**Authors:** Lu Zhao, Chao Wang, Dandan Qi, Meng Sun, Hong Qi, Yin Dong, Yunqing Zeng, Lele Tang, Jie Ding, Yudan Zhu, Qian Xiao, Wei Wu, Yonghua Ji, Jie Tao

**Affiliations:** 1 Shanghai Putuo Central School of Clinical Medicine, Anhui Medical University, Shanghai, China; 2 Nanxiang Branch of Ruijin Hospital, Shanghai Jiao Tong University School of Medicine, Shanghai, China; 3 Putuo Hospital, Shanghai University of Traditional Chinese Medicine, Shanghai, China; 4 Joint Laboratory of Nanxiang Branch of Ruijin Hospital-School of Life Sciences, Shanghai University, Shanghai, China; 5 Department of Pharmacy, The first Affiliated Hospital of Fujian Medical University, Fuzhou, China; 6 Department of Pharmacy, National Regional Medical Center, Binhai Campus of the First Affiliated Hospital, Fujian Medical University, Fuzhou, China

**Keywords:** BmK AS, epilepsy, kainic acid-induced epilepsy model, Nav1.6 sodium channel, neuroinflammation, pyroptosis

## Abstract

**Introduction:**

Voltage-gated sodium channel (VGSC) dysregulation, particularly of the Nav1.6 subtype, is a core mechanism underlying epileptogenesis and its associated neuropsychiatric comorbidities. The scorpion venom peptide BmK AS has demonstrated anticonvulsant potential, but its efficacy in chronic epilepsy and the precise mechanisms of action remain undefined.

**Methods:**

Here, we show that BmK AS exerts robust anti-epileptic and neuroprotective effects through converging mechanisms. In a kainic acid-induced mouse model, BmK AS treatment reduced mortality and seizure parameters. Electrophysiological studies assessed BmK AS modulation of VGSC subtypes. The functional relevance of Nav1.6 targeting was confirmed by the loss of BmK AS’s anti-seizure efficacy upon its pharmacological blockade in a PTZ-induced model. Furthermore, in both KA-induced chronic epilepsy models and native hippocampal neurons, BmK AS was evaluated for neuronal hyperexcitability and NLRP1 inflammasome-mediated pyroptosis.

**Results:**

BmK AS reduced mortality to 0% (vs. 40% in the model group) and significantly reduced seizure duration by 10.5% and the frequency of severe (stages 4 and 5) seizures by 68.8%. It also improved cognitive and psychiatric outcomes, significantly reversing epilepsy-associated spatial memory deficits and anxiety-/depression-like behaviors. Electrophysiological studies show that BmK AS nonlinearly inhibited multiple VGSC subtypes, with pronounced potency against Nav1.6, reducing the peak sodium current to 43% of control at 5 nM. BmK AS attenuated neuronal hyperexcitability and suppressed neuroinflammation by inhibiting the NLRP1 inflammasome pathway and the associated pyroptosis.

**Discussion:**

Our findings establish BmK AS as a promising multimechanistic therapeutic candidate, highlighting the strategic value of dual therapeutic actions, namely, Nav1.6 modulation and neuroinflammation inhibition, for epilepsy treatment.

## 1 Introduction

Epilepsy is a chronic neurological disorder characterized by transient cerebral dysfunction and has clinical manifestations of recurrent epileptic seizures. Afflicting approximately 70 million individuals globally, epilepsy represents a substantial burden on the nervous system and is associated with psychiatric comorbidities in 30%–50% of patients (Mula et al., 2022; Casale et al., 2021), with Nav1.6 dysfunction accounting for approximately 20% of epilepsy cases (Talwar and Hammer, 2021). The etiology of epilepsy is multifactorial, encompassing diverse factors such as central nervous system infections, perinatal injuries, and head trauma. If the seizures remain refractory, they may lead to irreversible neuronal damage (Toledo et al., 2025; Huang et al., 2021; Chen et al., 2024). Uncontrolled epilepsy can lead to serious complications, including mental confusion and cognitive decline (Sen et al., 2020; Ammothumkandy et al., 2025), which profoundly compromise the activities of daily living. Currently, management strategies are broadly categorized into pharmacotherapy and surgery. Owing to the limited applicability and inherent risks of surgical interventions, antiepileptic drugs remain the mainstay of treatment for the majority of patients. However, while effective in suppressing seizures, long-term administration of first-line agents is frequently associated with the development of drug tolerance and a spectrum of adverse effects, ranging from visual disturbances and dermatological reactions to systemic complications such as hepatotoxicity, pancreatitis, and renal impairment (Akyuz et al., 2021; Vezzani et al., 2022).

Genome-wide association studies (GWAS) in epilepsy have revealed a nucleotide substitution in the voltage-gated sodium channel (VGSC) gene *SCN8A*, which encodes the Nav1.6 channel. As pivotal members of the ion channel superfamily, VGSCs are transmembrane complexes that are indispensable for initiating and propagating action potentials, thereby driving epileptogenesis. Structurally, the channel complex is composed of a principal α-subunit that confers the core ion pore functionality and auxiliary β-subunits that fine-tune its gating properties, kinetics, and membrane density. Consequently, this sophisticated architecture ensures its stable presence on the cell membrane, thus facilitating its critical role in neuronal excitability (Barbieri et al., 2023).

The α-subunit is a 260-kDa polypeptide of approximately 2,000 amino acids, organized into four homologous domains (DI–DIV). Each domain consists of six transmembrane segments (S1–S6). The pore loop between S5 and S6 forms the ion conduction pathway, while the S4 segment serves as a voltage sensor with multiple positively charged residues (Barbieri et al., 2023). The β-subunit, a 33-kDa to 36-kDa protein with a single transmembrane domain, associates with the α-subunit via noncovalent interactions or disulfide bonds (Catterall, 2017). In the central nervous system, VGSC α-subunits are primarily represented by the Nav1.1 (*SCN1A*), Nav1.2 (*SCN2A*), Nav1.3 (*SCN3A*), and Nav1.6 (*SCN8A*) subtypes. A notable developmental shift in their expression is observed. Nav1.3 is predominantly expressed during the embryonic and neonatal stages, implicating its role in neurodevelopment. Conversely, Nav1.1, Nav1.2, and Nav1.6 are the predominant isoforms in the adult brain, underscoring their critical functions in mature neuronal circuitry (Catterall et al., 2005; Thouta et al., 2022). The Nav1.6 channel is abundantly expressed in the central and peripheral nervous systems, with prominent somatodendritic localization observed in the projection neurons of the hippocampus, cerebral cortex, and cerebellum regions. Notably, its expression is significantly upregulated in animal models of temporal lobe epilepsy (TLE) (Hargus et al., 2011). A well-defined functional property of Nav1.6 is its mediation of both the persistent (I_Na_P) and resurgent (I_Na_R) sodium currents. Convincing evidence further indicates that I_Na_P exerts a pathogenic influence, thereby linking this specific channel function directly to the process of epileptogenesis (Vera and Lippmann, 2021; Stafstrom, 2007). Therefore, targeted modulation of VGSCs, particularly the Nav1.6 subtype, represents a rational therapeutic strategy for epilepsy.

In this context, animal venom-derived peptides, particularly from scorpions, have garnered significant interest as high-affinity and subtype-selective modulators of VGSCs. Several scorpion venom peptides have demonstrated anticonvulsant potential as they interact with distinct extracellular receptor sites on VGSCs. For instance, TsTX from *Tityus serrulatus* is characterized as an α-type toxin that binds to site-3 of sodium channels in a voltage-dependent manner, suppressing channel inactivation and inducing spontaneous recurrent seizures in animal models, which establishes it as a useful tool in epilepsy research (Qu et al., 2026; Zhao et al., 2008). Notably, some β-toxins from the same venom, such as TsTX-I, bind to a different site (site-4) and alter channel activation (Sandoval and Lebrun, 2003). In addition, BmK IT2, a peptide derived from *Buthus martensii* Karsch, has been reported to exhibit dose-dependent anticonvulsant effects in rat models of epilepsy (Sandoval and Lebrun, 2002), and a recent study has suggested that it may alleviate seizures by targeting the Nav1.6 channel (Teixeira et al., 2010). Among these neuroactive peptides, BmK AS, a 66-amino-acid peptide stabilized by four disulfide bonds, derived from the venom of *Buthus martensii* Karsch (Ji et al., 1999), is classified as a “β-toxin with α-like properties” (Blumenfeld et al., 2009). This unique classification implies a novel mechanism: it binds to sites associated with β-toxins but can bidirectionally modulate sodium-channel gating. This bidirectional action—potentially suppressing inactivation at lower concentrations while facilitating it at higher concentrations—may allow for a more nuanced suppression of pathological hyperactivity, thus presenting a distinct pharmacological profile (Uzair et al., 2018). However, the efficacy of BmK AS in chronic epilepsy models, its subtype selectivity toward the clinically relevant Nav1.6, and its potential neuroprotective properties beyond electrophysiological modulation remain largely unexplored.

Prior investigations have established BmK AS as a bioactive scorpion venom peptide with modulatory effects on VGSCs. Studies have demonstrated its anticonvulsant efficacy in acute pentylenetetrazol (PTZ)-induced seizure models (Zhao et al., 2011) and characterized its complex, concentration-dependent modulation of recombinant Nav1.2 and Nav1.3 channels in heterologous expression systems (Liu et al., 2012; Zhu et al., 2009). However, several critical questions remain unanswered: (1) whether BmK AS exerts sustained therapeutic effects in *chronic* epilepsy models, which help better recapitulate the progressive nature of the disorder; (2) the extent of its selectivity and functional necessity for the epilepsy-implicated Nav1.6 subtype *in vivo*; (3) whether its mechanism extends beyond electrophysiological modulation to encompass the neuroinflammatory component that is integral to epileptogenesis. Consequently, this study aims to systematically investigate the anti-seizure and neuroprotective effects of BmK AS in a chronic kainic acid (KC)-induced epilepsy model. We hypothesize that its therapeutic benefits are mediated through a dual mechanism encompassing both the selective modulation of the Nav1.6 channel to normalize neuronal excitability and the suppression of neuroinflammation via the NLRP1 inflammasome–pyroptosis pathway.

## 2 Results

### 2.1 BmK AS reduces mortality and seizure severity in a kainic acid-induced murine model of epilepsy

The dose of 0.5 μg BmK AS was selected for intracerebroventricular administration in order to achieve local hippocampal concentrations within the low nanomolar concentration range that have been demonstrated to be sufficient for potent and selective inhibition of Nav1.6 channels, as demonstrated by electrophysiological data. A cohort of 10 C57BL/6 mice was designated as the control group (n = 10). Epilepsy was induced in 40 additional mice through intraperitoneal injection of KA (30 mg/kg, adjusted for body weight). Following administration, mice were immediately placed in individual observation chambers for continuous monitoring of seizure development. Behavioral seizures progressed sequentially through characteristic stages: initial hypoactivity, followed by perioral twitching, head nodding, tail hyperextension, tonic posturing, clonic convulsions, and, finally, jumping. The inclusion criteria for a successful model are as follows: mice that exhibit seizure severity reaching at least Racine stage IV or persistent stage-II seizures lasting longer than 60 min. Among the 40 KA-treated mice, two nonresponders and eight fatalities were excluded, leading to 30 validated epileptic models. These mice were then randomly allocated into three experimental groups (n = 10 per group): the KA+Saline group (i.p.), the KA+BmK AS (0.5 μg) group, and the KA+VPA (valproic acid, i.p., positive control) group.

Twenty-one days post-KA induction, the groups received their respective treatments. Video monitoring during the subsequent 2-h period revealed a mortality rate of 40% (four out of 10 mice) in the KA+Saline group, which was significantly higher than that in the KA+BmK AS group (0%, zero out of 10) and the KA+VPA group (10%, one out of 10). Detailed analysis of the seizure behavior parameters is summarized in Table 1. Compared to the KA+Saline group, treatment with BmK AS significantly prolonged seizure onset latency (by 5.2%, *P* < 0.01) and reduced seizure duration (by 2.5%, *P* < 0.01). Furthermore, BmK AS treatment markedly decreased the number of seizures across all Racine stages, with significant reductions observed in stages 1 and 2 (by 11.2%, *P* < 0.05), stage 3 (by 2.6%, *P* < 0.05), and stages 4 and 5 seizures (by 68.8%, *P* < 0.01).

**TABLE 1:** BmK AS suppresses KA-induced seizure severity.

Treatment	KA + Saline	KA + BmK AS	KA + VPA
Animal number (n)	10	10	10
Mortality	4	0	1
Latency(s)	79.5 ± 4.30	93.6 ± 22.70**	86.7 ± 11.38**/#
Seizure duration(s)	125.8 ± 2.98	122.6 ± 9.49**	116.5 ± 5.83**/#
Seizure number	​	​	​
Stage 1 and 2 seizures	19.7 ± 2.02	17.5 ± 5.2*	15.8 ± 2.60*/ns
Stage 3 seizures	3.8 ± 0.83	3.0 ± 1.13*	2.6 ± 0.33*/ns
Stage 4 and 5 seizures	3.2 ± 0.46	1.0 ± 0.57**	2.1 ± 0.40**/#

KA + BmK AS or KA + VPA vs. KA + Saline: *P < 0.05, **P < 0.01, ***P < 0.001, and ****P < 0.0001 by one-way ANOVA and *post hoc* LSD tests; KA + BmK AS vs. KA + VPA: #*P* < 0.05, ##*P* < 0.01, ###*P* < 0.001, and ####*P* < 0.0001 by one-way ANOVA with *post hoc* LSD tests; ns: non-significant.

When compared to the positive control valproic acid (VPA), BmK AS showed a comparable anti-seizure profile on some parameters but distinct effects on others. Both compounds significantly reduced the number seizures of stages 1, 2, and 3 to a similar extent, with no statistically significant difference observed between the two treatment groups (Table 1). However, significant differences were noted between the BmK AS and VPA groups in seizure latency, seizure duration, and the number of severe (stages 4 and 5) seizures (Table 1). Notably, BmK AS treatment resulted in a greater prolongation of seizure onset latency (17.7% increase vs. 9.1% in the VPA group) and a more pronounced reduction in the frequency of severe seizures (68.8% decrease vs. 34.4% in the VPA group), whereas VPA led to a greater reduction in the overall seizure duration (7.4% decrease vs. 2.5% in the BmK AS group).

### 2.2 BmK AS suppresses hippocampal local field potential (LFP) epileptiform activity in a KA-induced murine epilepsy model

**FIGURE 1:**
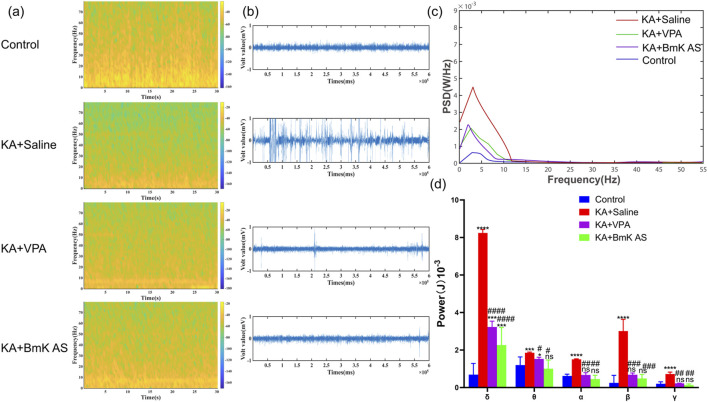
BmK AS suppresses hippocampal epileptiform activity in mice. **(a)** Spectral density heatmap of field potential activity in each group. **(b)** LFP recording of epileptic waveforms in each group. **(c)** Average power spectral density (PSD) maps in each group. **(d)** Statistical comparisons of PSDs for δ, θ, β, and γ bands in each group (n = 5 mice per group). Treatment groups vs. control group: *P < 0.05, **P < 0.01, ***P < 0.001, and ****P < 0.0001 by one-way ANOVA, ns: non-significant; KA + BmK AS group or KA + VPA group vs. KA + Saline group: #*P* < 0.05, ##*P* < 0.01, ###*P* < 0.001, and ####*P* < 0.0001 by one-way ANOVA with *post hoc* LSD tests.

LFP recordings were obtained from epileptic model mice across experimental groups using implanted cortical electrodes. Signal acquisition and analysis were performed with MATLAB R2023b (MathWorks) following established protocols. Quantitative comparisons demonstrated significant suppression of epileptiform field potentials in BmK AS-treated and VPA-treated mice relative to the model group (Figures 1a,b). Spectral analysis revealed decreased average power density in the treatment group, with statistically significant reductions in rhythmic energy across five characteristic frequency bands (Figure 1d). These results confirm the potent anti-seizure efficacy of BmK AS in modulating aberrant neuronal synchronization.

### 2.3 BmK AS attenuates KA-induced learning and memory deficits in epileptic mice

**FIGURE 2:**
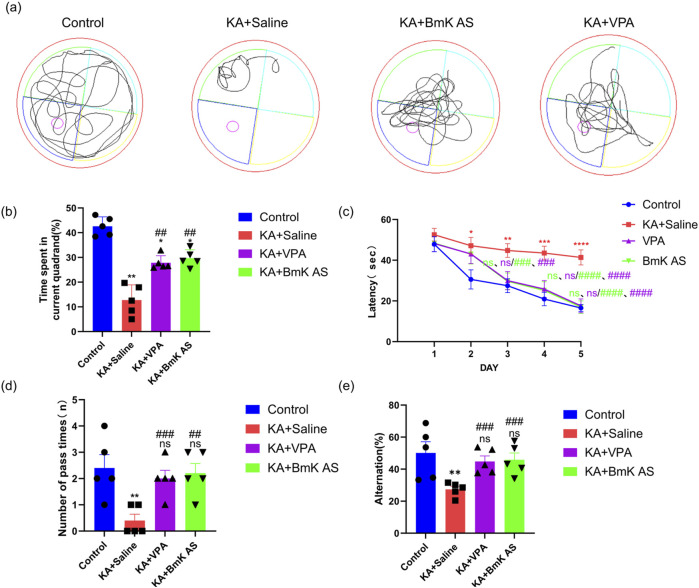
Effects of Bmk AS on learning and memory abilities in mice. **(a)** Swimming paths of mice in the water maze across groups. **(b)** Time spent in the target quadrant by each group of mice. **(c)** Escape latency of mice in each group during water maze training. **(d)** Number of platform crossings in the water maze by each group of mice. **(e)** Spontaneous alternation rates of mice in each group (n = 5 mice per group). Data represent mean ± SEM. Treatment groups vs. control group: *P < 0.05, **P < 0.01, ***P < 0.001, and ****P < 0.0001 by one-way ANOVA, ns: non-significant; KA + BmK AS group or KA + VPA group vs. KA + Saline group: #*P* < 0.05, ##*P* < 0.01, ###*P* < 0.001, and ####*P* < 0.0001 by one-way ANOVA with *post hoc* LSD tests.

To analyze the therapeutic potential of BmK AS against post-epileptic cognitive deficits, four experimental cohorts were established: naive controls (Control), epileptic models (KA+Saline), BmK AS-treated epileptic models (KA+BmK AS), and valproate sodium-treated epileptic models (KA+VPA). Cognitive functions were systematically evaluated using the Morris water maze (MWM) and Y-maze behavioral paradigms. In MWM place navigation trials, KA-induced epileptic mice exhibited significantly prolonged escape latency versus controls (*P* < 0.05, Figure 2c), whereas BmK AS treatment attenuated this impairment from day 3 (33.64 ± 2.65 s vs. 46.32 ± 2.20 s, *P* < 0.05, Figure 2c), achieving maximal efficacy on day 4 (28.37 ± 1.73 s vs. 45.64 ± 3.12 s, *P* < 0.01, Figure 2c) and day 5 (19.27 ± 2.51 s vs. 46.30 ± 5.08 s, *P* < 0.0001, Figure 2c).The KA+VPA group showed a similar improvement pattern. Spatial probe testing revealed that KA+Saline mice displayed reduced target quadrant dwell time (by 71.43%, *P* < 0.01, Figure 2b) and platform crossings (by 80.00%, *P* < 0.01, Figure 2d) compared to controls; thus, deficits were significantly reversed by BmK AS intervention. Furthermore, Y-maze assessment of working memory demonstrated that spontaneous alternation rates, a hallmark of spatial memory integrity, were markedly suppressed in KA+Saline mice (by 52.00%, *P* < 0.01, Figure 2e), with BmK AS treatment restoring spontaneous alternation to near-baseline levels (*P* < 0.001 vs. KA+Saline, Figure 2e). Furthermore, these improvements were comparable to those induced by the clinical anticonvulsant VPA. Collectively, these findings provide robust evidence that BmK AS ameliorates epilepsy-associated cognitive dysfunction through enhancement of both spatial learning and working memory capacities.

### 2.4 BmK AS modulates spontaneous exploration and alleviates anxiety-/depression-like phenotypes in a KA-induced murine epilepsy model

**FIGURE 3:**
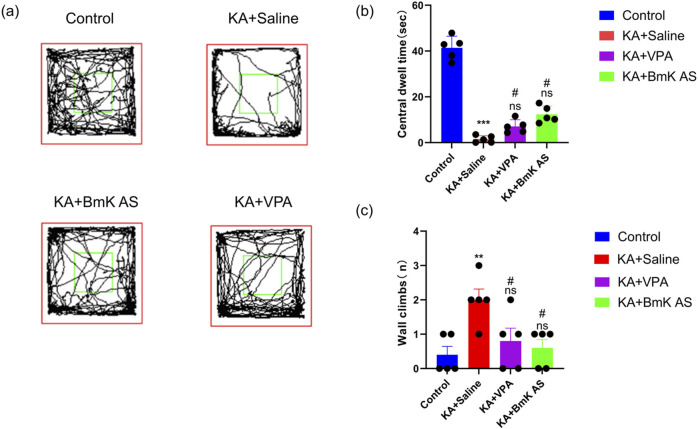
Effects of Bmk AS on anxiety- and depression-like behaviors in mice. **(a)** Representative exploration trajectories of mice across experimental groups. **(b)** Quantification of dwelling time in the central zone. **(c)** Wall climbing frequency (n = 5 mice per group). Treatment groups vs. control group: *P < 0.05, **P < 0.01, ***P < 0.001, and ****P < 0.0001 by one-way ANOVA, ns: non-significant; KA + BmK AS group or KA + VPA group vs. KA + saline group: #*P* < 0.05, ##*P* < 0.01, ###*P* < 0.001, and ####*P* < 0.0001 by one-way ANOVA with *post hoc* LSD tests.

The open-field test was performed to assess spontaneous locomotor activity and anxiety-/depression-like behaviors in KA-induced epileptic mice following BmK AS treatment. Key behavioral parameters, namely, time spent in the central zone and the frequency of wall climbing, were quantified. Compared with the Control group, the KA-treated mice exhibited a significant reduction in the time spent in the central zone (by 95.24%, *P* < 0.001, Figure 3b) and an increase in wall-climbing frequency (by 400.00%, *P* < 0.01, Figure 3c). Administration of BmK AS significantly decreased these alterations, increasing the time spent in the central zone (by 350.00%, *P* < 0.05 vs. KA group, Figure 3b) and reducing wall-climbing frequency (by 62.50%, *P* < 0.05 vs. KA group, Figure 3c), and these improvements were comparable to those induced by the clinical anticonvulsant VPA. These findings indicate that BmK AS treatment attenuates anxiety-/depression-like behaviors in epileptic mice.

### 2.5 Bidirectional modulation of BmK AS on I_Na_ amplitude at different concentrations

To quantify the direct effects of BmK AS on VGSC function, we performed whole-cell voltage-clamp recordings on HEK293T cells that stably express different VGSC α-subunits (rNav1.4, rNav1.5, mNav1.6, and hNav1.7). The cells were exposed to cumulative concentrations of BmK AS (5, 20, 100, 500, and 2,000 nM), with each concentration perfused for 5 min before measurement (see Methods 4.12 for details). The peak transient sodium current (I_Na_T) was elicited by a step depolarization to 0 mV from a holding potential of −80 mV. The current amplitudes were normalized to each cell’s own control (pre-toxin) value for analysis.

Rationale for channel selection: The four VGSC α-subtypes were selected based on their physiological relevance and utility in toxin characterization studies: rNav1.4 (rat skeletal muscle) and rNav1.5 (rat cardiac) are canonical subtypes for comparative pharmacology of sodium-channel toxins; mNav1.6 (mouse neuronal) is a CNS-enriched subtype that is strongly implicated in epileptogenesis, representing a key therapeutic target in our study; hNav1.7 (human peripheral neuronal) is a well-studied subtype crucial in pain signaling and is known for its sensitivity to various β-scorpion toxins, providing a reference for BmK AS’s potential broader pharmacological profile. Although we acknowledge that a direct comparison of orthologous channels (e.g., human vs. rodent Nav1.6) would be ideal for translational extrapolation, the selected panel allows for an initial assessment of BmK AS’s subtype selectivity and mechanism across functionally distinct channels.

**FIGURE 4:**
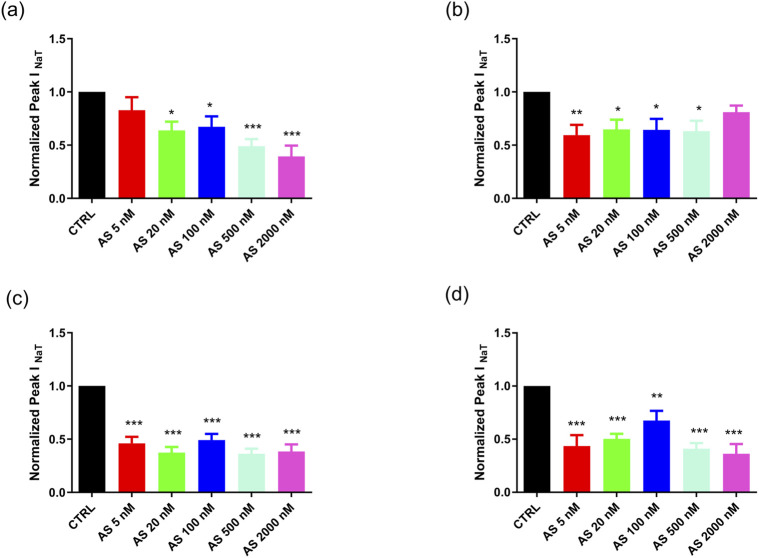
Inhibitory effect of BmK AS on the current amplitude of VGSC subtypes. **(a–d)** Normalized value of rNav1.4 **(a)**, rNav1.5 **(b)**, mNav1.6 **(c)**, hNav1.7 **(d)**, and peak I_Na_T in HEK293t cells in the presence of 5, 20, 100, 500, and 2,000 nM BmK AS. *P < 0.05, **P < 0.01, and ***P < 0.001; significant difference between the control and BmK AS; one-way ANOVA.

Application of BmK AS resulted in a significant inhibition of peak I_Na_T across all four VGSC subtypes, with a marked effect observed even at the lowest concentration tested (5 nM), where inhibition ranged from 25.6% (rNav1.4) to 42.9% (mNav1.6) (Figures 4a–d). The concentration–response relationship varied among the subtypes. For rNav1.4 and hNav1.7, the inhibition progressively increased with concentration, reaching a maximal blockade of 63.2% and 59.1%, respectively, at 2,000 nM. In contrast, the inhibitory effect on mNav1.6 appeared to saturate at low concentrations, with 5 nM producing 42.9% inhibition and higher concentrations (20 nM–2,000 nM) yielding only a modest additional effect (maximum 48.1% at 20 nM). For rNav1.5, the inhibition showed a nonmonotonic pattern, with 37.8% blockade at 5 nM, a slight decrease at 20 nM (31.1%), and a maximal effect of 40.0% at 500 nM before declining to 25.6% at 2,000 nM.

Furthermore, a comparison across subtypes at a low, physiologically relevant concentration (20 nM) indicated that BmK AS exhibited a relatively higher inhibitory effect on mNav1.6 (48.1%) compared to rNav1.4 (37.7%), rNav1.5 (31.1%), and hNav1.7 (38.6%), indicating a relatively preferential sensitivity of this epilepsy-associated subtype at low concentrations.

### 2.6 Modulation effect of BmK AS on the voltage-dependent activation of VGSCs

To investigate how BmK AS affects channel opening, we analyzed its effects on the voltage-dependence of activation, which determines the threshold and sensitivity for action potential initiation. The voltage-dependent activation was assessed by fitting conductance–voltage (G–V) relationships, derived from current–voltage (I–V) curves, to a Boltzmann function to obtain the half-maximal activation voltage (V_1/2_) and slope factor (k).

**FIGURE 5:**
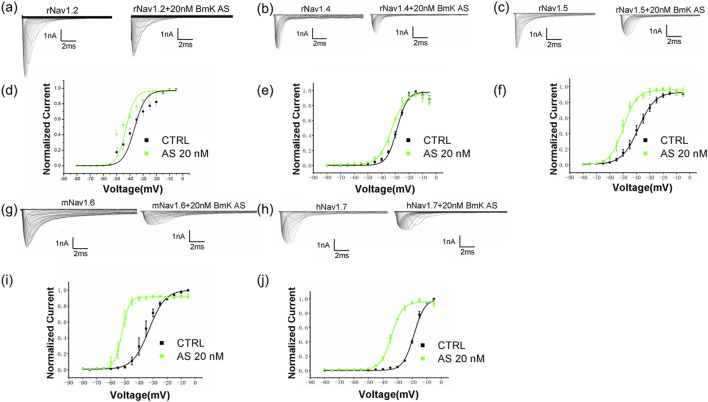
BmK AS differentially modulates the voltage dependence of sodium-channel activation across VGSC subtypes. **(a–c,g,h)** Representative sodium current traces of the indicated VGSC subtypes in the presence of 20 nM BmK AS. **(d–f,i,j)** Corresponding conductance–voltage (G–V) relationships (steady-state activation) for rNav1.2 **(d)**, rNav1.4 **(e)**, rNav1.5 **(f)**, mNav1.6 **(i)**, and hNav1.7 **(j)** derived from the data from current–voltage (I–V) relationships (n ≥ 6). The bidirectional, concentration-dependent modulation of mNav1.6 activation—with a shift toward more hyperpolarized potentials at 20 nM and a shift toward more depolarized potentials at 2 μM—is detailed in Results 2.6 and presented in Supplementary Table S3.

BmK AS differentially modulates the activation of VGSC subtypes. For rNav1.4, 20 nM BmK AS induced a significant shift of the activation curve toward more hyperpolarized potentials, with V_1/2_ shifting by approximately 10 mV to a more negative value (CTRL: V_1/2_ = −26.3 ± 0.85 mV, n = 13; 20 nM: V_1/2_ = −36.3 ± 0.79 mV, n = 8; Supplementary Table S1). A similar shift toward more hyperpolarized potentials was observed for rNav1.5, where 20 nM BmK AS shifted V_1/2_ by approximately 3 mV (CTRL: V_1/2_ = −41.2 ± 0.54 mV, n = 25; 20 nM: V_1/2_ = −45.6 ± 0.72 mV, n = 8; Figure 5c; Supplementary Table S2).

The modulation of mNav1.6 was particularly notable for its bidirectional concentration dependence. While 20 nM BmK AS caused a 6.2-mV shift of V_1/2_ toward more hyperpolarized potentials, a high concentration (2 µM) paradoxically shifted V_1/2_ by 4.9 mV toward more depolarized potentials (CTRL: V_1/2_ = −41.6 ± 0.48 mV, n = 15; 20 nM: V_1/2_ = −47.8 ± 0.96 mV, n = 6; 2 µM: V_1/2_ = −36.7 ± 0.93 mV, n = 8; Figure 5g; Supplementary Table S3). This bidirectional, concentration-dependent modulation of activation voltage dependence was a distinctive feature observed for mNav1.6 as the other tested subtypes (rNav1.4, rNav1.5, and hNav1.7) exhibited monophasic hyperpolarizing shifts across the same concentration range (Supplementary Tables S1, S2, S4).

For hNav1.7, BmK AS also induced shifts of V_1/2_ toward more hyperpolarized potentials across concentrations, with the magnitude of shift varying from 5.5 to 11.1 mV (CTRL: V_1/2_ = −28.8 ± 1.71 mV, n = 14; 5 nM: V_1/2_ = −39.3 ± 1.09 mV; 2,000 nM: V_1/2_ = −36.5 ± 0.94 mV; Supplementary Table S4). The slope factor (k) of the activation curve was increased by BmK AS (2,000 nM) for hNav1.7, but decreased for mNav1.6 at certain concentrations (Supplementary Tables S3, S4), indicating subtype-specific alterations in voltage sensitivity.

### 2.7 Modulation effect of BmK AS on voltage-dependent inactivation of VGSCs

Channel inactivation governs refractory periods and excitability. We analyzed BmK AS’s effects on steady-state fast inactivation and slow inactivation.

BmK AS (500 nM) significantly shifted the V_1/2_ of steady-state inactivation of rNav1.5 by 7.5 mV toward more hyperpolarized potentials (CTRL: V_1/2_ = −75.4 ± 1.53 mV, n = 25; 500 nM: V_1/2_ = −82.1 ± 2.01 mV, n = 6; Supplementary Table S2; Supplementary Figure S1b), with no significant effects on the other subtypes (Supplementary Figures S1a, c, d).

Analysis of fast inactivation revealed subtype-specific effects. BmK AS (5 nM) shifted the V_1/2_ of fast inactivation of rNav1.5 by 5.8 mV toward more hyperpolarized potentials (CTRL: V_1/2_ = −46.4 ± 1.01 mV, n = 25; 5 nM: V _1/2_ = −52.2 ± 1.28 mV, n = 11; Supplementary Table S2; Supplementary Figure S1f). In contrast, 500 nM BmK AS caused a shift toward more depolarized potentials in the fast inactivation of mNav1.6 (CTRL: V_1/2_ = −53.3 ± 1.30 mV, n = 15; 500 nM: V_1/2_ = −46.0 ± 1.92 mV, n = 8; Supplementary Table S3; Supplementary Figure S1g), while it shifted the fast inactivation of hNav1.7 by 9.8 mV toward more hyperpolarized potentials (CTRL: V_1/2_ = −26.7 ± 0.52 mV, n = 14; 500 nM: V_1/2_ = −36.5 ± 1.93 mV, n = 7; Supplementary Table S4; Supplementary Figure S1h). No significant effect was observed on rNav1.4 (Supplementary Figure S1e).

For slow inactivation, BmK AS (500 nM) caused a shift toward more depolarized potentials for rNav1.4 but a large shift toward more hyperpolarized potentials for rNav1.5 and mNav1.6 (Supplementary Tables S1–S3; Supplementary Figures S1i–k). The slow inactivation of hNav1.7 was not significantly affected (Supplementary Table S4; Supplementary Figure S1l).

These shifts in voltage-dependent inactivation have distinct implications for neuronal excitability. A shift toward more hyperpolarized potentials (e.g., fast inactivation of rNav1.5 and hNav1.7 and slow inactivation of rNav1.5 and mNav1.6) indicates that channels inactivate at lower membrane potentials, which would reduce the neuronal excitability by limiting the availability of sodium channels for subsequent action potentials. Conversely, the shift toward more depolarized potentials observed for fast inactivation of mNav1.6 at 500 nM would delay inactivation, potentially increasing excitability; however, this effect may be counterbalanced by the more potent inhibition of the peak current amplitude at low concentrations (Figure 4c) and the overall anti-epileptic outcome observed *in vivo*.

### 2.8 Effects of BmK AS on inactivation kinetics of VGSCs

Gating kinetics influence the action potential waveform and firing frequency. We analyzed the effects of BmK AS on the inactivation time course and recovery from inactivation.

BmK AS slowed the inactivation kinetics of several subtypes. It increased the inactivation time constant (τ) of rNav1.4 (at 100 and 2,000 nM), rNav1.5 (at 5 and 20 nM), and mNav1.6 (at 5, 500, and 2,000 nM) (Supplementary Figures S2a–c). The inactivation kinetics of hNav1.7 were not significantly altered (Supplementary Figure S2d).

BmK AS also affected recovery from inactivation. It significantly increased the recovery time constant (τrec) of rNav1.4 (at 2,000 nM), rNav1.5 (at 20 nM), and hNav1.7 (at 2,000 nM), while slightly reducing the percentage of channels that fully recovered (Supplementary Figure S3; Supplementary Tables S1, S2, S4). The recovery kinetics of mNav1.6 were not significantly altered (Supplementary Figure S3c; Supplementary Table S3).

Slowing of inactivation kinetics prolongs the open state of sodium channels, which could theoretically increase sodium influx and excitability. However, this effect is likely outweighed by the concomitant reduction in peak current amplitude (Figure 4) and the shifts in inactivation voltage dependence described above. The net effect on neuronal firing will depend on the integration of multiple modulatory actions.

### 2.9 Modulation effect of BmK AS on the recovery from inactivation of VGSCs

For assessing kinetic correlations between inactivation and recovery from inactivation of VGSCs, recovery kinetics were examined on HEK293T VGSCs. The time constant of recovery underlying rNav1.4 was increased in the presence of 2,000 nM BmK AS (CTRL: τrec = 1.5 ± 0.10 ms, n = 13; 2,000 nM: τrec = 3.6 ± 0.27 ms, n = 8; Supplementary Table S1), and the percentage recovery in rNav1.4 decreased from 98% ± 0.39% for the control to 96% ± 0.11% in the presence of 2,000 nM BmK AS (Supplementary Figure S3a). The time constant of recovery underlying rNav1.5 was also increased in the presence of 20 nM BmK AS (CTRL: τrec = 2.5 ± 0.08 ms, n = 25; 20 nM: τrec = 4.0 ± 0.27 ms, n = 8; Supplementary Table S2), and the percentage recovery in rNav1.5 decreased from 98% ± 0.45% for the control to 97% ± 0.10% in the presence of 20 nM BmK AS (Supplementary Figure S3b). No significant differences between the control group and BmK AS groups were found in the time constant of mNav1.6 (Supplementary Figure S3c). The time constant of recovery underlying hNav1.7 was increased in the presence of 2,000 nM BmK AS (CTRL: τrec = 1.8 ± 0.07 ms, n = 14; 2,000 nM: τrec = 3.9 ± 0.18 ms, n = 7; Supplementary Table S4), and the percentage recovery in hNav1.7 decreased from 96% ± 0.70% for the control to 95% ± 1.12% in the presence of 2,000 nM BmK AS (Supplementary Figure S3d).

Prolongation of recovery from inactivation means that channels require more time to return to the closed state after an action potential, thereby limiting the maximum firing frequency. This effect, observed for rNav1.4, rNav1.5, and hNav1.7 at certain concentrations, would contribute to a use-dependent reduction in neuronal excitability, which is beneficial for seizure suppression. The lack of effect on mNav1.6 recovery kinetics indicates that this particular gating property is not a major contributor to the anti-epileptic action on this subtype, where the peak current inhibition and modulation of activation/inactivation voltage dependence may perform more prominent functions.

### 2.10 Key binding sites of Nav1.6 for BmK AS identified by docking simulations

A structural homology model of Nav1.6 was generated using Discovery Studio 2018, followed by molecular docking simulation with BmK AS to predict the key interacting amino acid residues (Supplementary Table S5; Supplementary Figure S4). The simulation revealed 10 potential binding interactions: CYS 46 of BmK AS with TYR 1191 of Nav1.6 Domain III, ASN 63 of BmK AS with ARG 599 of Domain II, TYR 38 of BmK AS with SER 1177 of Domain III, GLY 64 of BmK AS with PHE 598 of Domain II, CYS 62 of BmK AS with PHE 598 of Domain II, ASN 2 of BmK AS with TYR 1191 of Domain III, TYR 57 of BmK AS with ILE 1198 of Domain III, GLY 3 of BmK AS with TYR 1191 of Domain III, and PHE 39 of BmK AS with LYS 1179 of Domain III. These interactions indicate the potential key contact sites between BmK AS and Nav1.6.

### 2.11 Effects of BmK AS on the firing properties of hippocampal slice neurons

**FIGURE 6:**
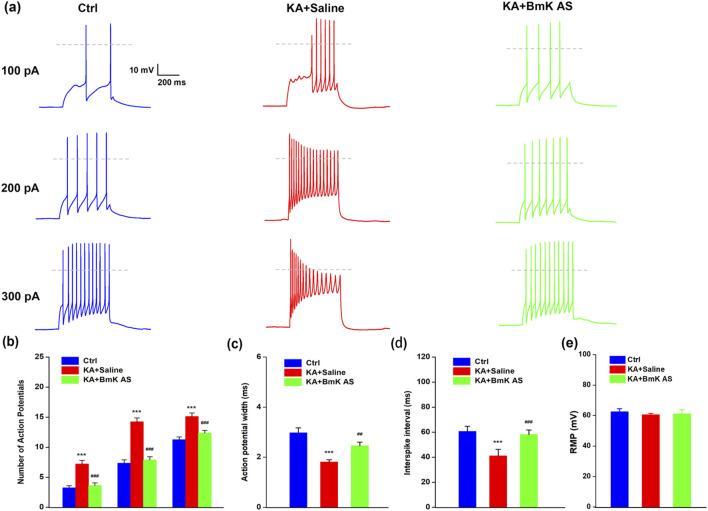
BmK AS reduced kindling-induced neuronal hyperexcitability in hippocampal slices. **(a)** Action potential (AP) responses to current injection by hippocampal pyramidal neurons from CTRL (black), KA + saline (red), and KA + BmK AS (green) group mice. The 0-mV level is indicated by the dashed line. **(b)** Average number of APs elicited at each current level. **(c)** AP width calculated at one-half peak height during 300-pA current injection. **(d)** Interspike interval preceding the ninth AP during a 300-pA current injection. **(e)** Resting membrane potential (RMP) of CA1 neurons from each experimental group. No significant difference was observed between groups (one-way ANOVA, P > 0.05). Data are presented as mean ± SEM (n ≥ 6 neurons per group). Control group vs. KA group or KA + BmK AS group: ***P < 0.001; KA + BmK AS group vs. KA group: #*P* < 0.05, ##*P* < 0.01, ###*P* < 0.001, and ####*P* < 0.0001 by one-way ANOVA.

KA induces elevated neuronal firing in hippocampal circuits, which is a key contributor to the kindling effect. To determine whether BmK AS could mitigate this hyperexcitability, action potential (AP) responses to incremental current injections (100, 200, and 300 pA) were assessed in hippocampal slices obtained from control (CTRL, non-kindled), saline-treated-kindled, and BmK AS-treated-kindled mice (Figure 6a). All recordings were performed under a clamped resting membrane potential of −80 mV to ensure consistent baseline conditions across the groups.

We first compared the passive membrane properties of CA1 pyramidal neurons across the experimental groups. The resting membrane potential (RMP) was not significantly different between the groups (CTRL: −64.3 ± 1.2 mV; KA+Saline: −62.9 ± 1.4 mV; KA+BmK AS: −62.1 ± 1.3 mV; P > 0.05, one-way ANOVA), indicating that BmK AS treatment did not alter the basal membrane potential (Figure 6e). Compared to CTRL neurons, those from saline-treated-kindled mice fired a significantly higher number of APs across all current steps. This heightened excitability was effectively suppressed by BmK AS treatment as the number of evoked APs in the BmK AS group was significantly reduced and did not differ from the CTRL levels (Figure 6b). In contrast, the AP width at half-amplitude remained unchanged across all three groups (Figure 6c). Further analysis of the firing patterns revealed that neurons from saline-treated kindled mice exhibited markedly impaired spike-frequency accommodation, resulting in significantly shorter interspike intervals and higher sustained discharge frequencies compared to the CTRL. Treatment with BmK AS restored this adaptive property, as evidenced by the significantly longer interspike intervals compared to the saline group (Figure 6d). These results collectively demonstrate that BmK AS attenuates KA-induced hyperexcitability during kindling by reducing the overall spike output and preserving spike-frequency accommodation, thereby limiting sustained high-frequency firing in hippocampal neurons.

### 2.12 BmK AS inhibits voltage-gated sodium currents in primary cultured hippocampal neurons

**FIGURE 7:**
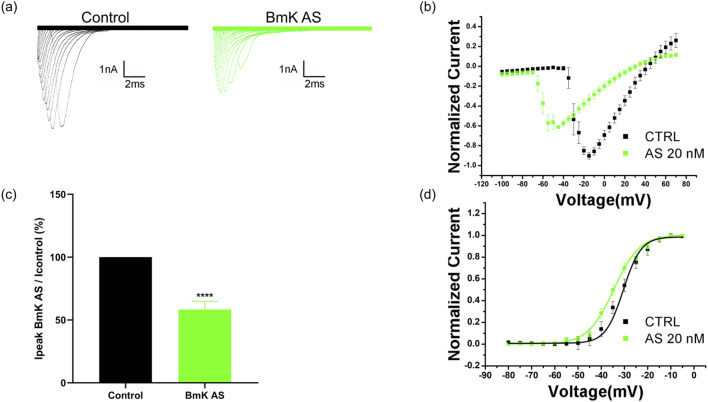
BmK AS directly inhibited sodium currents in hippocampal neurons. **(a)** Sodium currents in hippocampal neurons before and after direct administration of BmK AS. **(b)** Current–voltage relationship for peak sodium current. **(c)** Effects of BmK AS administration on the amplitude of sodium currents in hippocampal neurons (n = 6 neurons per condition, control group vs. BmK AS group: ****P < 0.0001, paired t-test). Effects of BmK AS administration on the amplitude of sodium currents in hippocampal neurons. **(d)** Normalized G–V curves (mean ± SEM, n = 6) with Boltzmann fits.

To validate the modulatory effects of BmK AS on native neuronal VGSCs in a more physiological context, whole-cell patch-clamp recordings were performed on primary cultured hippocampal neurons. Figure 7a shows the representative sodium current (I_Na_) traces in hippocampal neurons before and directly after the application of BmK AS. Bath application of 20 nM BmK AS led to a marked reduction in the peak I_Na_ amplitude. Statistical analysis across multiple neurons confirmed that BmK AS (20 nM) significantly reduced the peak I_Na_ amplitude to 57.0% ± 3.2% of the pre-application baseline levels (*P* < 0.0001, n = 6 neurons, Figure 7c). The I–V relationship of the peak I_Na_ was examined before and after BmK AS application (Figure 7b). BmK AS treatment reduced the peak current amplitude across the entire voltage range.

To determine whether BmK AS alters the voltage sensitivity of channel activation, we constructed normalized G–V curves (Figure 7d). Boltzmann fitting of individual cell data yielded the half-activation voltage (V_1_/_2_) −30.7 ± 1.03 mV in control neurons and −34.5 ± 1.82 mV in BmK AS-treated neurons. Statistical analysis revealed no significant difference between the groups (paired t-test, P > 0.05, n = 6). These results demonstrate that BmK AS effectively inhibits VGSCs in native hippocampal neurons without significantly modifying their activation voltage dependence. This finding corroborates the direct action of BmK AS on neuronal VGSCs and supports its potential to modulate neuronal excitability at the cellular level.

### 2.13 A selective Nav1.6 inhibitor mimics the anti-seizure effects of BmK AS in a PTZ-induced seizure model

To further investigate the role of Nav1.6 in mediating the anti-seizure effects of BmK AS, we used a PTZ-induced acute seizure model. Although the KA-induced chronic epilepsy model (used in previous sections) recapitulates the progressive nature of temporal lobe epilepsy, the PTZ model is widely used for rapid screening of anticonvulsant agents and allows direct comparison with a selective Nav1.6 inhibitor (zandatrigine) in a well-established acute seizure paradigm.

Seizure parameters were compared among four experimental groups: PTZ+Saline, PTZ+BmK AS, PTZ+VPA, and PTZ+Nav1.6 inhibitor (zandatrigine). Mice were administered PTZ by intraperitoneal (i.p.) injection at a sub-convulsive dose for kindling. Thirty minutes prior to PTZ injection, mice in the PTZ+Nav1.6 inhibitor group received an i.p. injection of the selective Nav1.6 inhibitor zandatrigine (50 mg/kg). Immediately following PTZ (60 mg/kg) administration, mice received an intra-cerebroventricular (i.c.v.) injection of either BmK AS (0.5 µg), VPA (i.p., positive control), or saline and then a second i.p. PTZ injection after 2 h (for kindling).

As shown in Table 2, the second PTZ injection induced fatal seizures in the saline group (two of 10 mice, 20%), while no deaths were observed in the BmK AS or Nav1.6 inhibitor groups (zero of 10 mice, 0%). Compared to the PTZ+Saline group, treatment with BmK AS significantly prolonged the seizure onset latency (by 146%, *P* < 0.0001), reduced seizure duration (by 45%, *P* < 0.0001), and decreased the number of seizures across all Racine stages, with significant reductions observed in stages 1 and 2 (by 49%, *P* < 0.01), stage 3 (by 39%, *P* < 0.05), and stages 4 and 5 (by 66%, *P* < 0.0001). The positive control VPA also exhibited significant anti-convulsive effects across all parameters (Table 2). A key finding was that pharmacological inhibition of Nav1.6 alone with zandatrigine produced significant anti-seizure effects, which were comparable to those produced by BmK AS and VPA. In the PTZ+Nav1.6 inhibitor group, the protective effects on seizure latency, duration, and severity showed no statistically significant difference compared to those in either the PTZ+BmK AS or PTZ+VPA groups, while all three treatment groups showed significant differences from the PTZ+Saline group. These results show that pharmacological blockade of Nav1.6 alone with zandatrigine significantly attenuated PTZ-induced seizures across multiple parameters, phenocopying the anti-seizure efficacy of BmK AS (Table 2). In most of the seizure parameters measured—including latency, duration, and stages 1, 2, and 3 seizures—the effects of BmK AS were not statistically distinguishable from those of the Nav1.6 inhibitor. This finding provides indirect evidence supporting the involvement of Nav1.6 in the therapeutic mechanism of BmK AS. Nonetheless, we acknowledge that definitive proof of Nav1.6 necessity would require additivity experiments, which remains a limitation of the current study.

**TABLE 2:** Effect of BmK AS and Nav1.6 inhibition on PTZ-induced seizures.

Treatment	PTZ + Saline	PTZ + Nav1.6 inhibition	PTZ + BmK AS	PTZ + VPA
Animal number (n)	10	10	10	10
Mortality	2	0	0	0
Latency(s)	106.22 ± 7.38	254.36 ± 8.31****	261.32 ± 28.41****/ns	223.96 ± 21.26****/##
Seizure duration(s)	295.32 ± 16.87	160.56 ± 12.38****	162.25 ± 16.83****/ns	158.64 ± 10.62****/ns
Seizure number	​	​	​	​
Stage 1 and 2 seizures	11.63 ± 2.21	5.82 ± 1.16**	5.82 ± 0.62**/ns	5.54 ± 0.83**/ns
Stage 3 seizures	9.27 ± 1.26	5.06 ± 1.03*	5.63 ± 0.92*/ns	5.77 ± 0.86*/ns
Stage 4 and 5 seizures	11.86 ± 2.30	5.64 ± 1.83****	4.00 ± 0.62****/#	4.28 ± 0.69****/#

PTZ+Nav1.6 Inhibition, PTZ + BmK AS or PTZ + VPA vs. PTZ + Saline: *P < 0.05, **P < 0.01, ***P < 0.001, and ****P < 0.0001 by one-way ANOVA and *post hoc* LSD tests; ns: non-significant. PTZ + BmK AS or PTZ + VPA vs. PTZ+Nav1.6 Inhibition: #*P* < 0.05, ##*P* < 0.01, ###*P* < 0.001, and ####*P* < 0.0001 by one-way ANOVA and *post hoc* LSD tests; ns: non-significant.

### 2.14 A selective Nav1.6 inhibitor mimics the suppressive effects of BmK AS on PTZ-induced epileptiform activity

**FIGURE 8:**
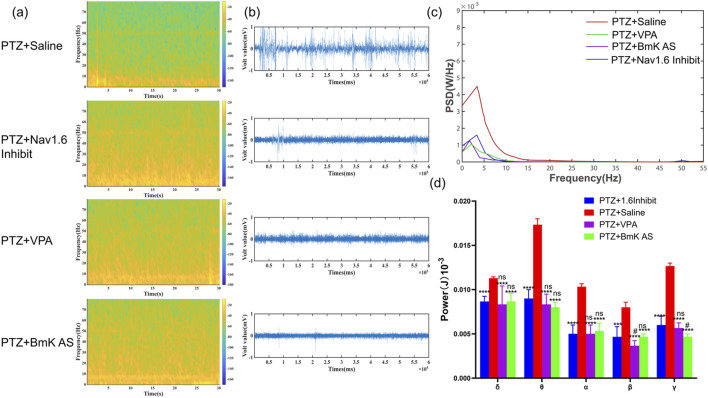
A selective Nav1.6 inhibitor mimics the suppressive effects of BmK AS on PTZ-induced hippocampal epileptiform activity. **(a)** Spectral density heatmap of field potential activity in each group. **(b)** LFP recording of epileptic waveforms in each group. **(c)** Average power spectral density (PSD) maps in each group. **(d)** Statistical comparisons of PSDs for δ, θ, β, and γ bands in each group (n = 10 mice per group). PTZ+Nav1.6 Inhibition, PTZ + BmK AS or PTZ + VPA vs. PTZ + Saline: *P < 0.05, **P < 0.01, ***P < 0.001, and ****P < 0.0001 by one-way ANOVA and *post hoc* LSD tests, ns: non-significant. PTZ + BmK AS or PTZ + VPA vs. PTZ+Nav1.6 Inhibition: #*P* < 0.05, ##*P* < 0.01, ###*P* < 0.001, and ####*P* < 0.0001 by one-way ANOVA and *post hoc* LSD tests, ns, non-significant.

To determine whether the functional integrity of Nav1.6 channels is required for suppression of epileptiform activity by BmK AS, local field potential (LFP) recordings were compared among PTZ+Saline (control group), PTZ+BmK AS, PTZ+VPA, and PTZ+Nav1.6 inhibitor groups during PTZ-induced seizures. The effect of PTZ on LFP activity was visualized by constructing power spectral density (PSD) plots. High-frequency, large-amplitude spikes were observed in the LFP recording of PTZ-injected mice that were administered saline (Figures 8a,b). A key finding was that the administration of the Nav1.6 inhibitor group significantly suppressed these epileptiform signals, and its effect was comparable to that of BmK AS and VPA (Figures 8a,b). The PSD plot revealed high-amplitude discharges in the low-frequency (δ) band of saline-injected mice that were significantly diminished by BmK AS treatment (Figure 8d). The PSD analysis further revealed that the power of all five frequency bands (δ, θ, α, β, and γ, Figure 8d) was significantly elevated in the saline group following PTZ injection. Administration of BmK AS significantly suppressed these epileptic waveforms as there were significant differences compared to that in the saline group (δ wave, *P* < 0.01, n = 10; θ wave, *P* < 0.001, n = 1; γ wave, *P* < 0.001, n = 10). Importantly, the power in these bands in the PTZ+Nav1.6 inhibitor group showed no statistically significant difference from that in the PTZ+BmK AS or PTZ+VPA groups, while all three treatment groups showed significant differences from the PTZ+Saline group. These results demonstrate that pharmacological inhibition of Nav1.6 alone with zandatrigine significantly suppressed PTZ-induced epileptiform activity, phenocopying the effects of BmK AS in most frequency bands (Figure 8). Specifically, in the δ, θ, and β bands, the suppressive effects of BmK AS were not statistically distinguishable from those of the Nav1.6 inhibitor. However, in the γ band, BmK AS showed a more pronounced suppression compared to the Nav1.6 inhibitor alone (Figure 8d). This finding provides indirect evidence supporting the involvement of Nav1.6 in the anti-epileptic mechanism of BmK AS, while also suggesting that additional mechanisms may contribute to its effects on high-frequency oscillations. As noted in Section 2.13, definitive proof of Nav1.6 necessity would require additivity experiments, which remains a limitation of the current study.

### 2.15 BmK AS attenuates NLRP1-mediated pyroptosis in hippocampal neurons

**FIGURE 9:**
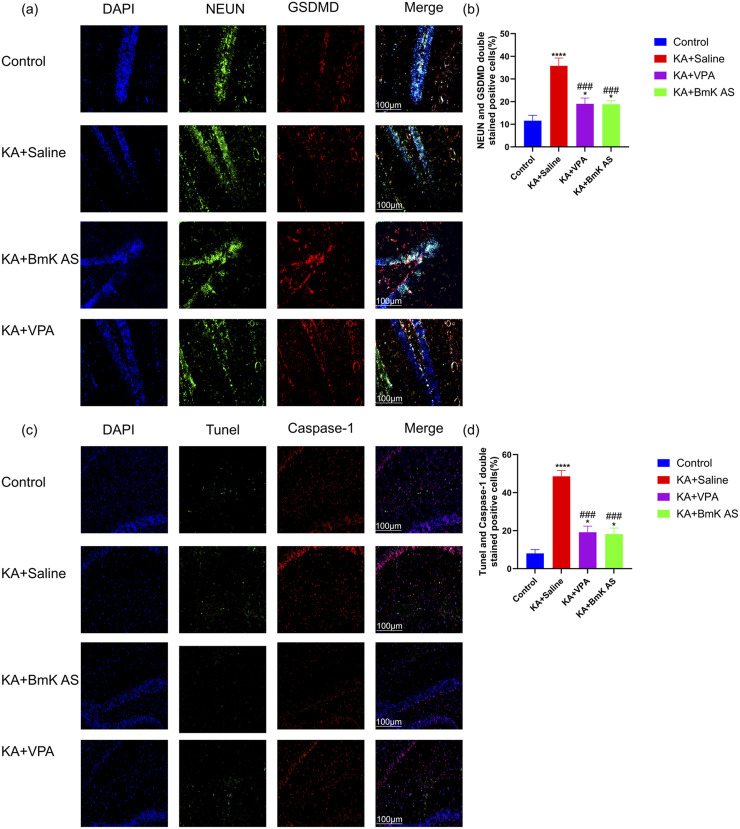
BmK AS attenuates KA-induced neuronal pyroptosis in epileptic mice. **(a)** Neun/GSDM double-labeling in cortical neurons. **(b)** Quantification of NeuN/GSDMD-co-labeled cells in different experimental groups. **(c)** TUNEL/caspase-co-staining in hippocampal tissue. **(d)** Quantification of TUNEL/caspase-co-labeled cells in different experimental groups (n = 5 mice per group). Treatment groups vs. control group: *P < 0.05, **P < 0.01, ***P < 0.001, and ****P < 0.0001 by one-way ANOVA, ns, non-significant; KA + BmK AS group or KA + VPA group vs. KA + Saline group: #*P* < 0.05, ##*P* < 0.01, ###*P* < 0.001, and ####*P* < 0.0001 by one-way ANOVA with *post hoc* LSD tests.

To investigate the role of BmK AS in modulating pyroptosis, dual immunofluorescence staining was performed on frozen brain sections from four experimental groups: Control, KA + Saline (model group), KA + BmK AS, and KA + VPA. Compared with the Control group, KA-induced epileptic mice showed a significant increase in the proportion of GSDMD/NeuN double-positive neurons (by 27%) and in the colocalization of TUNEL with caspase-1 (CASP1) in the hippocampal regions (by 14%) (Figures 9b,d), indicating enhanced pyroptotic cell death during epileptogenesis. In contrast, both the KA + BmK AS and KA + VPA groups exhibited a reduced proportion of GSDMD/NeuN colocalized neurons and fewer TUNEL/CASP1 double-positive cells relative to the model group. Furthermore, quantitative analysis confirmed that the number of GSDMD/NeuN double-positive neurons was significantly lower in the KA + BmK AS and KA + VPA groups (Figure 9b), demonstrating that BmK AS effectively attenuates neuronal pyroptosis in the epileptic brain.

**FIGURE 10:**
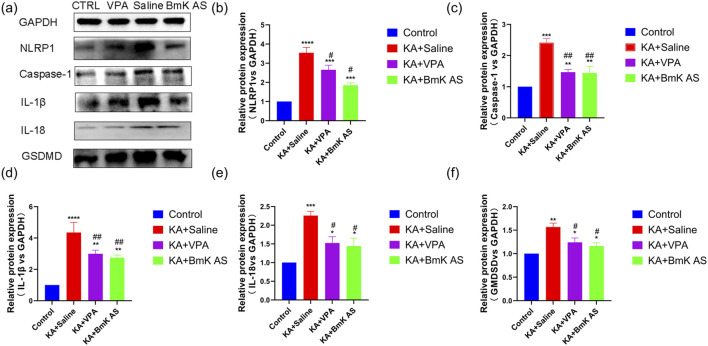
BmK AS attenuates pyroptosis and inflammation in KA-induced epileptic mice. **(a)** Representative Western blot bands of NLRP1, caspase-1, GSDMD, IL-1β, and IL-18 in hippocampal tissues. **(b–f)** Quantitative analysis of protein expression levels: NLRP1, caspase-1, and GSDM; IL-1β and IL-18 (n = 5 mice per group). Treatment groups vs. control group: *P < 0.05, **P < 0.01, ***P < 0.001, and ****P < 0.0001 by one-way ANOVA, ns, non-significant; KA + BmK AS group or KA + VPA group vs. KA + Saline group: #*P* < 0.05, ##*P* < 0.01, ###*P* < 0.001, and ####*P* < 0.0001 by one-way ANOVA with *post hoc* LSD tests.

Western blot analysis revealed significant differences in pyroptosis-related protein expression (NLRP1, caspase-1, and GSDMD) and inflammatory cytokines (IL-18 and IL-1β) across the experimental groups. Compared to those from the control group, hippocampal tissues from chronic KA-induced epileptic mice exhibited markedly elevated levels of NLRP1 (*P* < 0.0001), caspase-1 (*P* < 0.001), and GSDMD (*P* < 0.01) (Figures 10b,c,f). Concurrently, the expressions of inflammatory cytokines IL-18 (*P* < 0.001) and IL-1β (*P* < 0.0001) were significantly upregulated (Figures 10d,e). BmK AS treatment attenuated these effects, with the BmK AS-treated group or VPA showing reduced NLRP1 (*P* ≤ 0.001; Figure 10b), caspase-1 (*P* < 0.01; Figure 10c), and GSDMD (*P* ≤ 0.05; Figure 10f) expressions. Similarly, IL-1β (*P* < 0.01) and IL-18 (*P* < 0.05) levels were significantly decreased in the BmK AS-treated group or VPA compared to the epilepsy model (Figure 10e). These data collectively demonstrate that BmK AS suppresses both pyroptosis-associated pathways and neuroinflammatory responses in chronic epilepsy.

## 3 Discussion

Our study demonstrates that BmK AS exerts significant anti-epileptic and neuroprotective effects in both a KA-induced chronic epilepsy model and a PTZ-induced acute seizure model, as evidenced by reduced mortality, attenuated seizure severity, improved cognitive performance, and inhibition of epileptiform activity (Tables 1 and 2; Figures 1–3, 8). The mechanistic basis for these effects involves dual pathways: direct modulation of a key VGSC subtype, thereby regulating neuronal hyperexcitability, and subsequent suppression of neuroinflammatory signaling.

The potent anti-epileptic efficacy of BmK AS can be quantitatively and logically linked to the strength of its dual molecular actions. First, its substantial inhibition of voltage-gated I_Na_, with notable potency against the epileptogenesis-relevant Nav1.6 subtype, directly dampens neuronal membrane excitability and curtails the capacity for pathological high-frequency firing—the core electrophysiological substrate of seizures. Second, its robust inhibition of key pyroptosis and inflammatory markers (e.g., NLRP1, caspase-1, and IL-1β) alleviates neuroinflammation, thereby disrupting the inflammation-mediated increase in neuronal excitability and epileptic network formation. This “dual-pathway” strategy—simultaneously targeting electrophysiological hyperexcitability and neuroinflammatory signaling—aligns with the design philosophy of multitarget anti-seizure agents (such as other venom-derived peptides including BmK IT2) and likely produces synergistic therapeutic effects.

Compared to many conventional anti-seizure medications (e.g., sodium-channel blockers such as carbamazepine or levetiracetam) that primarily modulate a single electrophysiological mechanism, the dual mechanism of BmK AS offers distinct potential advantages: (1) Synergistic efficacy: By co-targeting the coexisting “electrical storm” and “inflammatory milieu” in epilepsy pathology, BmK AS may yield stronger effects in refractory cases or epilepsy types with significant neuroinflammation. (2) Potential disease-modifying effects: Through inhibiting inflammatory pathways such as NLRP1, it may slow epileptogenesis, reduce neuronal damage, and extend its effects beyond mere symptom control. (3) A potentially improved safety window: The synergistic action of the dual pathways might allow for effective seizure inhibition at lower doses, potentially reducing the risks of side effects associated with high inhibition of a single target. Therefore, BmK AS represents a more comprehensive and promising therapeutic strategy for epilepsy.

Prior studies have laid important groundwork for understanding BmK AS as a VGSC modulator. It has been characterized as a sodium-channel site 4-selective modulator with dose-dependent anticonvulsant activity in acute PTZ-induced seizures, although with limited efficacy in pilocarpine models, indicating model-dependent actions (Zhao et al., 2011). Electrophysiological analyses in heterologous systems revealed its ability to modulate various subtypes: BmK AS facilitates activation and inhibits slow inactivation of Nav1.3 (Liu et al., 2012) and exerts a unique U-shaped, dose-dependent modulation on Nav1.2a, indicating potential binding to two distinct receptor sites (Zhu et al., 2009). These studies collectively established BmK AS as a complex peptide that is capable of bidirectional, concentration-dependent gating effects across several VGSC subtypes.

Building upon this foundation, our study significantly advances the field in several key aspects. First, we transition from acute to chronic epilepsy modeling, demonstrating that BmK AS provides sustained anti-seizure effects, reduces mortality, and, crucially, ameliorates long-term cognitive and psychiatric comorbidities in a KA-induced temporal-lobe epilepsy model—a dimension not addressed in prior studies on acute epilepsy (Zhao et al., 2011). Second, while earlier work highlighted the effects on Nav1.3 (Liu et al., 2012) and Nav1.2 (Zhu et al., 2009), we identify Nav1.6 as a key functional target *in vivo*. Our data not only show that BmK AS has higher potency and a unique bidirectional gating effect on Nav1.6 (Figures 4c, 5b,c) but also shows that a selective Nav1.6 inhibitor (zandatrigine) phenocopied the anti-seizure effects of BmK AS in the PTZ model (Table 2; Figure 8), providing indirect evidence for the involvement of Nav1.6 in its therapeutic mechanism. It should be noted, however, that definitive proof of Nav1.6 necessity would require additivity experiments (e.g., testing BmK AS in the presence of the inhibitor), which were not performed in the current study. Third, and most notably, we discover a previously unreported anti-neuroinflammatory mechanism of BmK AS—the inhibition of the NLRP1 inflammasome–pyroptosis pathway (Figures 9, 10). This represents a novel complementary action that is independent of its direct channel modulation, addressing a key pathological component in chronic epilepsy.

Electrophysiological analyses indicate that BmK AS functions as a potent modulator of VGSCs, with a relatively higher sensitivity toward Nav1.6, a subtype that is strongly implicated in epileptogenesis (Hargus et al., 2011; Blumenfeld et al., 2009; Hargus et al., 2013). While BmK AS modulated the gating of all tested VGSC subtypes (Figures 4, 5; Supplementary Figures S1–S3), its effects on Nav1.6 were particularly notable. At low nanomolar concentrations (5 nM–20 nM), BmK AS produced a more pronounced inhibition of Nav1.6 peak current compared to its effects on other subtypes such as rNav1.5 at the same concentrations (Figures 4b,c). Furthermore, Nav1.6 exhibited a unique, concentration-dependent bidirectional modulation of activation voltage dependence that is not observed in the other subtypes under the same conditions (Figures 5b,c). This distinct pharmacological profile supports its effective engagement of neuronal Nav1.6 channels. This engagement is sufficient to significantly alter neuronal excitability, as validated by the toxin’s inhibition of I_Na_ in native hippocampal neurons (Figure 7). Critically, similar efficacy between BmK AS and a selective Nav1.6 inhibitor in the PTZ model (Table 2; Figure 8) further supports the functional relevance of this channel subtype.

At low concentrations, BmK AS inhibited Nav1.6 currents (Figure 4c) and caused a shift in the activation curve toward more hyperpolarized potentials (Figure 5b). While such a shift would theoretically enhance excitability by lowering the threshold for activation, this effect is outweighed by the pronounced reduction in the peak current amplitude, resulting in a net inhibition of neuronal firing (Figure 6). It is important to contextualize these findings within the unique pharmacology of scorpion β-toxins. As a β-toxin with α-like properties (Uzair et al., 2018), BmK AS does not function as a simple channel blocker. Instead, β-toxins bind to receptor site-4 on VGSCs and characteristically shift the voltage dependence of activation toward more hyperpolarized potentials, facilitating channel opening at lower thresholds (Cestèle and Catterall, 2000; Thomsen et al., 1995; Mata et al., 2018; Zhang et al., 2019). This “facilitation of activation,” when combined with a simultaneous reduction in the peak current amplitude (as observed in Figure 4), results in a net inhibition of pathological high-frequency firing—a mechanism often described as “early opening leading to early suppression” (Gurevitz, 2012; Abroug et al., 1995).These actions align with its classification as a scorpion β-toxin exhibiting α-type functional properties (Uzair et al., 2018), allowing an interaction with distinct neurotoxin receptor sites on VGSCs (Thomsen et al., 1995; Mata et al., 2018; Zhang et al., 2019; Gurevitz, 2012). The observed bidirectional modulation indicates that BmK AS may operate through two binding modes: a high-affinity interaction that impedes inactivation and a low-affinity interaction that promotes inactivation (Abroug et al., 1995).

Furthermore, we identified a previously uncharacterized property of BmK AS: robust inhibition of hippocampal NLRP1 inflammasome activation and associated pyroptosis in the chronic epilepsy (Figures 9, 10). Notably, although concurrent anti-epileptic effects (mediated via Nav1.6) and anti-pyroptotic effects were observed, our data do not support a direct causal link between Nav1.6 modulation and NLRP1 pathway inhibition. The molecular mechanism underlying the inhibition of pyroptosis by BmK AS is not fully elucidated and represents an important direction for future investigation.

Rather than a limitation, we propose that this dual pharmacological profile constitutes a key advantage of our study. Epileptogenesis is increasingly understood as a process driven by a vicious cycle between neuronal hyperexcitability and neuroinflammation (Birnbaum et al., 2017). While most current therapies primarily target excitability, we show that BmK AS concurrently addresses both core pathological processes: it reduces neuronal excitability via its potent and relatively selective modulation of Nav1.6 and independently attenuates neuroinflammatory damage by inhibiting the NLRP1-/caspase-1/GSDMD-mediated pyroptotic cascade. However, we acknowledge that our evidence for Nav1.6 necessity is indirect. While the similar efficacy between BmK AS and the selective Nav1.6 inhibitor zandatrigine (Table 2; Figure 8) strongly indicates that Nav1.6 is a primary downstream effector, this does not constitute a definitive proof of necessity. A rigorous test would require additivity experiments in which BmK AS is administered after Nav1.6 blockade to assess the residual effects. The absence of such data is a limitation of the present study. Nonetheless, the concordance between pharmacological inhibition and toxin action, together with the known pharmacology of β-toxins discussed above, strongly supports Nav1.6 as a key mediator of BmK AS’s anti-seizure effects. Whether these effects stem from a single primary target or reflect independent yet synergistic actions, the combined outcome establishes BmK AS as a novel multimechanistic therapeutic candidate. This dual-target engagement likely explains its superior performance in improving survival and cognitive recovery as it disrupts the disease process at two key nodes, thereby more effectively interrupting the pathogenic feedback loop. The previously reported antinociceptive effects of BmK AS, which are attributed to its modulation of VGSCs in primary sensory neurons (Chen et al., 2006), further validate the functional relevance of its ion channel targeting.

In summary, BmK AS represents a novel multitarget strategy for epilepsy treatment, uniquely combining the potent modulation of the VGSC Nav1.6—a key mediator of its anti-seizure effects supported by our findings—with the inhibition of NLRP1-dependent pyroptosis. This dual mechanism may offer therapeutic advantages over conventional antiepileptic drugs, which are often limited by efficacy gaps and adverse effects (Akyuz et al., 2021). Given the established role of VGSCs in pain signaling pathways (Shields et al., 2018; Lai et al., 2003), the pharmacological profile of BmK AS also supports its potential applicability in other neurological disorders. Future studies should aim to delineate its precise binding site on Nav1.6, identify the molecular target responsible for its anti-pyroptotic activity, and systematically evaluate its long-term safety profile.

## 4 Materials and methods

### 4.1 Animals

C57BL/6J mice were obtained from Shanghai Bikai Keyi Biotechnology Co., Ltd. [certification number: SCKK (Shanghai) 2018-0006]. All experimental procedures were performed in accordance with the National Institutes of Health (NIH) Guide for the Care and Use of Laboratory Animals. The animals were housed under specific pathogen-free (SPF) conditions with environmental parameters strictly controlled at 23 °C–25 °C and 60% relative humidity. A standardized 12-h light/dark cycle was maintained to ensure circadian rhythm synchronization. All surgery procedures and experimental protocols were approved by the Experimental Animal Ethics Committee of Shanghai Putuo District Central Hospital and conducted in accordance with relevant regulations and guidelines (license number: DWEC-A-202206003).

### 4.2 Surgical implantation of drug-delivery catheters and local field potential (LFP) electrodes in mice

Under Zoletil50 anesthesia, mice were fixed in a stereotaxic frame (Narishige, Tokyo, Japan). After shaving and disinfecting the scalp with betadine, a midline incision was made. The dura mater was carefully punctured and cleaned with hydrogen peroxide irrigation. Based on a mouse brain atlas, coordinates were determined for dual implantation: a drug-infusion guide cannula into the left lateral ventricle (AP: −0.3 mm, ML: +1.0 mm, DV: −2.0 mm from bregma) and bipolar LFP electrodes into the contralateral (right) hippocampal CA3 region (AP: −2.3 mm, ML: +2.5 mm, DV: −1.8 mm). A 1-mm^2^ craniotomy was drilled at each site using a micro-drill, and the implants were inserted vertically. Dental cement was applied to secure the devices upon reaching the target depth. Post-surgery, the animals were maintained on a 42 °C heating pad until full recovery (Yoshizaki et al., 2020).

### 4.3 Experimental design, drug administration, and seizure behavior analysis

#### 4.3.1 PTZ-induced acute seizure model

Forty mice were randomly assigned to four groups (n = 10/group): PTZ+Saline (i.p.), Nav 1.6 Inhibition Model+PTZ, PTZ+Valproic Acid (VPA), and PTZ+BmK AS. After a 1-h acclimatization period in a glass chamber (40 cm × 30 cm × 50 cm), the Nav 1.6 Inhibition Model group received an i.p. injection of zandatrigine (50 mg/kg) to suppress central Nav 1.6 channels. Subsequently, mice in the Nav 1.6 Inhibition Model, VPA, and BmK AS groups were injected i.p. with PTZ (60 mg/kg) to induce seizures, while the Control mice received an equal volume of 0.9% saline. Seizure behavior was immediately recorded for the assessment of (i) latency to the first stage-2 seizure (incubation period); (ii) duration and severity of each seizure according to the Racine scale (stage 0: freezing; I: rigid posture/tail; II: head nodding/sitting with forepaw tremor; III: rearing with occasional falls; IV: bilateral forelimb clonus with rearing/balance loss; V: repeated falling with tonic–clonic activity) (Fat et al., 1997); (iii) total number of seizures (Zhao et al., 2011). A seizure event was defined as convulsive activity bounded by quiescent intervals of ≥5 s. Following this baseline PTZ challenge, the VPA group received VPA (200 mg/kg, i.p.). Mice in the model and BmK AS groups were anesthetized with ether and administered either normal saline (model) or BmK AS (in 1 µL of 0.9% saline) via the pre-implanted cannula. Two hours later, all mice received a second PTZ injection (60 mg/kg, i.p.) and were monitored for 2 h. The anticonvulsant activity of BmK AS was evaluated by comparing the incubation period, total seizure duration, number of seizures, and maximum Racine score after the second PTZ injection against the baseline (first injection) values.

#### 4.3.2 KA-induced chronic temporal lobe epilepsy (TLE) model

Fifty C57BL/6 male mice were acclimated for 1 week and then randomly allocated to a TLE induction group (n = 40) or a Control group (n = 10). TLE was induced by a single i.p. injection of KA (30 mg/kg). Control mice received 0.9% saline. Mice were monitored for 1 h post-injection; seizure severity was scored using the Racine scale (Racine, 1972), and status epilepticus (SE) was defined as continuous stage >3 activity lasting >30 min.

Mice that developed chronic TLE were randomly divided into three treatment groups (n = 10/group): Model (saline, i.p.), VPA (positive control, 200 mg/kg/day, i.p.), and BmK AS (0.5 µg/day, intra-cerebroventricular). Treatments were administered once daily for 7 consecutive days, starting 3 days post-KA administration. Drug delivery in the model and BmK AS groups utilized the implanted cannula system described in Section 4.2. On day 28 post-KA administration, spontaneous epileptic behavior in each group was video-recorded for 6 h. Blinded analysis was used to determine the seizure grade, frequency, total duration of tonic spasms (stage ≥4), and total seizure burden (duration × number). Additionally, behavioral assessments (including the Morris water maze, Y-maze, and open-field tests) were conducted on separate animal subgroups (n = 5 per group) that did not undergo LFP electrode implantation to avoid potential confounding effects of the surgical procedure on long-term behavioral performance.

### 4.4 Electroencephalographic signal acquisition in murine models

The LFP signals of mice in each experimental group were recorded using the Plexon acquisition system. Two stainless-steel screws were placed in the anterior skull to serve as grounding points. A microwire electrode was positioned onto the dorsal surface of the right hippocampus using stereotaxic coordinates (AP: −2.2 ± 0.1 mm, ML: +0.8 ± 0.1 mm, DV: −1.4 ± 0.1 mm) according to a previously established protocol (Bartolomei et al., 2012). The guide cannula and microwire assembly were then fixed to the skull using dental acrylic. LFP signals were amplified using an OmniPlex system (Plexon, United States) and displayed with Offline Sorter V4 software. In brief, the head-mounted electrode connector was attached to a pre-amplifier interfaced with an analog-to-digital (A/D) converter. Raw signals were acquired under the following conditions: 1,000× gain (amplitude range approximately −2 to +2 V), band-pass filtering between 1.6 and 100 Hz, and a sampling frequency of 1,000 Hz. Each seizure recording session lasted at least 30 min. Recorded data were exported to MATLAB (MathWorks, United States) for spectral analysis. Signals were decomposed into standard frequency bands (δ: 0 Hz–4 Hz, θ: 4 Hz–8 Hz, α: 8 Hz–13 Hz, β: 13 Hz–30 Hz, γ: 30 Hz–100 Hz) by applying wavelet transforms, Welch’s method with a Hamming window, and fast Fourier transforms. Time-varying spectral power was subsequently calculated using the prescribed weighted formula:

$$\int_{-\infty }^{+\infty }x^{2}\left(t\right)dt=\frac{1}{2\pi }\int_{-\infty }^{+\infty }{\left|X\left(j\omega \right)\right|}^{2}d\omega .$$

$$P=\lim_{T\to \infty }\frac{1}{2\pi }\int_{-T}^{T}x^{2}\left(t\right)dt=\frac{1}{2\pi }\int_{-\infty }^{+\infty }\lim_{T\to \infty }\frac{1}{2\pi }{\left|X_{T}\left(\omega \right)\right|}^{2}d\omega .$$

Twenty-eight days following KA administration, all mice underwent 30-min LFP recordings after i.p. injection of PTZ. Raw EEG signals were exported via the OmniPlex system, and spectral analysis was performed using MATLAB R2023b. Statistical evaluation of spectral power values was carried out with Origin 9.0 software. Motor seizures were classified by two trained researchers, who were blinded to the experimental groups, based on synchronized LFP patterns and corresponding behavioral observations.

### 4.5 Morris water maze

The Morris water maze (MWM) (Morris, 1984) setup comprised a circular blue tank (150 cm diameter × 50 cm height) filled with water maintained at 25 °C (±1 °C). The water was rendered opaque by adding non-toxic white paint. The pool was surrounded by distinct spatial cues. A circular platform (10 cm in diameter) was placed in the center of one quadrant and submerged approximately 1.5 cm beneath the water surface. A ceiling-mounted video system recorded all swimming paths.

Following 2 weeks of drug administration, mice underwent 2 days of habituation, during which they swam freely in the pool for 60 s each day. Subsequently, the animals received four training trials per day over 4 consecutive days. Each trial began with the mouse released from a different starting point along the pool wall. The time taken to find and mount the hidden platform (escape latency) was manually recorded. Once the platform was located, the mouse remained on it for 60 s to reinforce spatial learning. If an animal failed to find the platform within 60 s, it was gently guided onto the platform and allowed to stay there for the same duration. On day 6, a probe test was conducted to assess spatial memory. The platform was removed, and each mouse was released from a novel starting point and allowed to swim for 60 s. An observer recorded the number of passes through the former platform location. Additionally, the time spent in the target quadrant (the quadrant that previously contained the platform) and the number of entries into this quadrant were later analyzed from video recordings using tracking software (Shanghai Jilian, Shanghai, China).

### 4.6 Y maze

The Y-maze apparatus (Shanghai JiLian Web Tech Ltd.) consists of three equidistant arms arranged at 120° angles to form a symmetrical configuration (Kraeuter et al., 2019a). In the initial session, one compartment was blocked while the mice could freely explore the two remaining arms for 5 min. Following a 30-min break, the mice were returned to the same starting arm for a subsequent session, in which they were permitted to explore all three arms freely for 10 min. During the second session, the movement paths, frequency of entries into specific arms, and time spent in novel versus familiar arms were examined using video-tracking software (Shanghai Jilian, Shanghai, China). All arms were wiped with alcohol between trials to eliminate residual odors. Successful alternations were recorded when the mouse consecutively visited all three distinct arms without repetition. The alternation percentage was calculated using the following formula:

$$\text{Alternation rate }\left(\%\right)=\left[\text{Number of correct alternations }/\;\left(\text{Total arm entries }‐\;2\right)\right]\times 100\%.$$

### 4.7 Open-field testing

The open-field test (OFT) serves as a well-established behavioral assay for quantifying anxiety-related responses in rodent models. The experimental apparatus consisted of a polyvinyl chloride chamber (42 cm × 42 cm × 42 cm) with distinct spatial divisions, where a central quadrant (20 cm × 20 cm × 20 cm) was operationally defined as the anxiogenic zone. Animal movement patterns were continuously monitored for 10 min using an automated video-tracking system (Stoelting Co., Wood Dale, IL, United States) with triaxial motion detection capabilities. Behavioral parameters were subsequently analyzed, including the total ambulatory distance, temporal duration in the central zone, and path length specifically within the central quadrant. Consistent with standardized anxiety assessment protocols, reduced central-zone occupancy time and decreased exploratory motility in the central region were interpreted as validated indices of anxiety-like behavioral manifestations (Zimcikova et al., 2017; Kraeuter et al., 2019b).

### 4.8 Immunofluorescence

Brain sections were retrieved from storage at −80 °C and equilibrated to room temperature for 30 min. The sections were rinsed three times in 0.01 M PBS for 5 min each, followed by perimeter delineation using a hydrophobic barrier pen. Permeabilization was performed with 0.5% PBST under gentle agitation for 30 min, after which the sections were washed three times with PBS for 5 min each. Antigen retrieval solution (Beyotime, Shanghai, China) was applied and incubated at room temperature for 40 min with maintenance of moisture to prevent drying, followed by three additional PBS washes. The blocking solution (Beyotime, Shanghai, China) was then incubated with the sections for 1 h at ambient temperature under controlled humidity. Primary antibodies (rabbit anti-Caspase-1/rabbit anti-GSDMD/mouse anti-NeuN, Abcam, United States) diluted at 1: 200 were applied and incubated overnight in a humidified chamber at 4 °C. The following day, the sections were equilibrated to room temperature for 30 min and subjected to four 5-min PBS washes. Fluorescent secondary antibodies (goat anti-rabbit/anti-mouse IgG, 1:200; Abcam, United States) were applied under light-protected conditions for 2 h, followed by three 10-min PBS washes. TUNEL detection solution (Beyotime, Shanghai, China) was incubated with the sections in the dark at room temperature for 60 min, after which three 10-min PBS washes were performed. Nuclear counterstaining was conducted using DAPI (1:10,000, Beyotime, Shanghai, China) for 10 min, followed by three final 10-min PBS washes. Sections were mounted in 50% glycerol-based medium and stored under light-protected conditions at 4 °C to preserve fluorescence integrity.

### 4.9 Western blotting

Hippocampal tissues were homogenized in RIPA lysis buffer (Yeasen, Shanghai, China) supplemented with PMSF protease inhibitor, followed by centrifugation at 12,000 × g for 10 min at 4 °C to collect the supernatant. The protein concentration was quantitatively determined using the bicinchoninic acid (BCA) assay (Yeasen, Shanghai, China). Subsequently, protein samples underwent thermal denaturation at 95 °C for 10 min and were stored at −20 °C for subsequent analysis. SDS-PAGE gels (MERCK, United States) with stacking and separating layers were prepared, and denatured protein samples were loaded for electrophoresis, initially at 70 V for 30 min followed by 120 V for 1 h to separate proteins by molecular weight. The resolved proteins were transferred to activated PVDF membranes (MERCK, United States), and nonspecific binding sites were blocked with 5% BSA. Membranes were cut according to the target protein molecular weights and incubated overnight at 4 °C with primary antibodies against NLRP1 (1:500), caspase-1 (1:1,000), GSDMD (1:1,000), IL-1β (1:500), and IL-18 (1:500) (Abcam, United States). After washing with TBST, membranes were incubated with HRP-conjugated secondary antibodies at room temperature, followed by additional TBST washes to remove the unbound antibodies. Protein bands were finally visualized using ECL chemiluminescence (Yeasen, Shanghai, China), and signals were captured with an imaging system.

### 4.10 Preparation of plasmids and reagents

The plasmids pEZ-Lv206-SCN9A (hNav1.7) and pcDNA3.1-SCN8A (mNav1.6) were donated by Professor Yang Shilong (College of Wildlife and Protected Area, Northeast Forestry University, Harbin 150040, China). The plasmids pcDNA3.1-SCN4A (rNav1.4) and pcDNA3.1-SCN5A (rNav1.5) were donated by Kaoru Yamaoka (Department of Physiology, School of Medicine, Hiroshima University).

The crude BmK venom was purchased from a scorpion culture farm in Henan Province, China. The venom was filtered with a Sephadex G-50 column and purified as described by Liu et al. The purity of the toxin was confirmed by mass spectrometry and peptide sequencing. Stock solutions of BmK AS (0.1 mM BmK AS plus 25 g·L^−1^ BSA) were dissolved in the bath solution to obtain the desired concentration before electrophysiological recording. Unless otherwise stated, all chemicals were obtained from Sigma.

### 4.11 Cell culture and transfection

The HEK293T cell line was obtained from Cell Bank of Shanghai Institutes for Biological Sciences, Chinese Academy of Sciences. The cells were cultured in Dulbecco’s modified Eagle medium (DMEM; Gibco, Invitrogen, Grand Island, NY, United States) supplemented with 2 mM L-glutamine and 10% heat-inactivated fetal bovine serum (FBS; Gibco, Invitrogen). Culture dishes were incubated at 37 °C in a humidified atmosphere containing 5% CO_2_ and subcultured approximately every 2–3 days.

First, the HEK293T cells were transferred to 24-well plates (Corning, United States). When the cells’ confluence reached 80%–90%, 3 µg–6 µg VGSC subtype (Nav1.4, 1.5, 1.6, or 1.7, respectively) plasmid per well and 0.25 µg pEGFP-N1 (Life Technologies, United States) per well by administering 1.5 µL P3000 and 1.5 µL Lipofectamine 3000 were co-transfected. Finally, HEK293T cells with green fluorescence of EGFP were selected for patch clamping (Zhang et al., 2020).

### 4.12 Whole-cell patch-clamp recordings in cultured cells (HEK293T and primary hippocampal neurons)

Whole-cell I_Na_ recordings were performed as described previously (John et al., 2004), using an EPC-10 amplifier (HEKA Elektronik, Lambrecht, Germany) at room temperature. Patch pipettes were fabricated from glass capillary tubes (VitalSense, China) by PC-10 Puller (Narishige, Tokyo, Japan) with a resistance of 2 MΩ–3 MΩ. The internal solution contained (in mM) 120 CsF, 10 HEPES, 10 EGTA, and 15 NaCl at pH = 7.25. The external solution contained (in mM) 135 NaCl, 10 HEPES, 1 MgCl_2_, 2 CaCl_2_, 10 glucose, and 5 KCl at pH = 7.4 (Sigma). The internal and external solutions were adjusted to osmolarities of 285 mOsm–290 mOsm and 295 mOsm–300 mOsm, respectively. The same solutions were used for recordings from primary hippocampal neurons.

Data acquisition and stimulation protocols were controlled by Patchmaster software (HEKA Elektronik). Capacitance transients and series resistance errors were compensated by 80%. Cells were discarded when the series resistance values were above 20 MΩ. Linear leakage was subtracted using the P/4 protocol. Data were sampled at 20 kHz and low-pass-filtered at 10 kHz. The rate of solution exchange was studied using solutions with different NaCl concentrations and found to be approximately 95% complete within 60 s. For pharmacological testing, BmK AS solution was applied locally to the recorded cell via a puff pipette (tip diameter 0.3 mm) using a gravity-driven perfusion system (ALA Scientific Instruments, United States). The pipette tip was positioned 125 μm–150 μm away from the cell, and the superfusion rate was maintained at 200 μL min^-1^–300 μL min^-1^. Sodium currents were recorded before and after exposure to BmK AS at concentrations of 5, 20, 100, 500, and 2,000 nM, with each concentration perfused for 5 min prior to recording and without a washout period.

During recordings, cells were held at −80 mV and depolarized in 10 mV steps from −100 to +50 mV for 200 ms per step. Series resistance compensation was applied in the range of 70%–85% to minimize voltage errors, and cells with uncompensated series resistance >10 MΩ were excluded from the analysis.

The peak currents were elicited by step pulses ranging from −120 to +50 mV for 100 ms, with increments of 5 mV. The I_Na_T was normalized for cell capacitance and plotted against voltage to generate the I–V curves. For calculating the voltage dependence of activation, the sodium conductance was obtained using the formula:

$$G\left(V\right)=I\left(V\right)/\;\left(V\;‐\text{Erna}\right),$$

where I(V) is voltage V corresponding to the I_Na_T and Erna is the reversal potential. It was then fitted to a Boltzmann equation:

$$G=\frac{I}{\;\left(1+\exp\left[\left(V\;‐\;V_{1/2}\right)/K\right]\right)},$$

where V_1/2_ is the voltage at which half-maximal activation occurs and k is the slope factor. The voltage-dependence of inactivation was analyzed by a tertiary exponential function to fit the decay course of I_Na_T:

$$f\left(t\right)=C+A1\exp\left[‐\left(t‐t0\right)/\;\tau 1\right]+A2\exp\left[‐\left(t‐t0\right)/\;\tau 2\right]+A3\exp\left[‐\left(t‐t0\right)/\;\tau 3\right].$$

Here, C is the steady-state asymptote approximating to the non-inactivation persistent I_Na_P, where t is time, t0 is the time at which the I_Na_ were just starting to decrease, and A1, A2, and A3 represent the amplitudes of channels inactivating with the time constants τ1, τ2, and τ3, respectively. The voltage dependence of fast and slow inactivation were analyzed by two-pulse protocols composed of a 10- or 100-ms pre-pulse, respectively, to potentials ranging from −140 to +20 mV in 10-mV steps by a test pulse of 0 mV for 60 ms. The amplitudes of the I_Na_ were normalized to their maximal value and plotted as channel availability versus prepulse potential. Data were fitted to a Boltzmann equation:

$$f\left(x\right)=\frac{\left(1‐C\right)}{\left\{1+\exp\left[\left(x‐V_{1/2}\right)/k\right]\right\}}+C,$$

where V_1/2_ is the voltage midpoint of the fast or slow inactivation, k is the slope factor, and C is the steady-state asymptote.

The time history of sodium channel reactivation from the inactive state was studied by the double-pulse method. The clamping potential of 293T cells was −140 mV. The initial stimulation voltage was 0 mV, and it lasted for 50 ms. At the end of stimulation, a 2-ms interval was maintained with a voltage of −140 mV. This was followed by −10 mV stimulation for 50 ms. The ratio of the sodium channel reactivation is determined by the I_Na_T amplitude of the second pulse divided by the I_Na_T amplitude of the first pulse. After stimulation, the voltage was restored to −70 mV. Then, it was repeated, increasing the interval by +2 ms each time and stopping when the interval is 40 ms. Recovery data were fitted with a single exponential equation of the form:

$$I/\text{Imax}=1‐A\times \exp\left(‐t/\tau \text{rec}\right),$$

where A is the relative proportion of current recovery and t is the recovery interval with the time constant τrec.

For primary hippocampal neurons, recordings were performed between 14 and 20 days *in vitro*. All electrophysiological protocols were identical to those used for HEK293T cells, and the effects of BmK AS were examined after 5-min perfusion.

### 4.13 Primary hippocampal neuron culture

Hippocampal tissues were dissected from E16–18 fetuses in ice-cold Dulbecco’s phosphate-buffered saline (DPBS). Following meningeal and vascular tissue removal under a dissection microscope, the tissue was minced and subjected to enzymatic digestion using 0.125% trypsin at 37 °C for 15 min with intermittent shaking every 5 min. After aspiration of the trypsin solution, the digestion was halted by adding 1 mL of the plating medium. The tissue suspension was combined with 1.5 mL of the plating medium and 0.1 mL of DNase I in a culture dish, gently triturated using a pipette, and allowed to settle for 2 min. The supernatant containing single cells was collected, transferred to a 15-mL conical tube, and centrifuged at 1,000 rpm for 5 min. The pelleted cells were resuspended and plated onto poly-D-lysine (0.1 mg mL^-1^)-coated 6-well plates at a density of 5 × 10^5^ cells per well. Cells were maintained in a humidified incubator at 37 °C with 5% CO_2_. After 4 h–6 h, the medium was replaced, and cultures were grown for 6 days with half-medium change on day 3. To suppress glial proliferation, 100 μL of 5-fluoro-2′-deoxyuridine (FUDR) was added to each well. Following one complete medium change, one-third of the medium was refreshed every 3 days.

### 4.14 Hippocampal slice preparation and current clamp recordings

Following deep anesthesia with isoflurane, mice were perfused transcardially with an ice-cold sucrose-containing saline solution (in mM: 124 NaCl, 2.5 KCl, 1.25 NaH_2_PO_4_, 26 NaHCO_3_, 2 CaCl_2_, and 10 D-glucose at pH 7.4). Brains were then rapidly extracted after decapitation. Transverse hippocampal slices (350 μm thick) were prepared using a VT1200S vibratome (Leica Microsystems Inc.). The slices underwent an initial 30-min recovery incubation in standard artificial cerebrospinal fluid (aCSF; in mM: 119 NaCl, 2.5 KCl, 1.2 NaH_2_PO_4_, 25 NaHCO_3_, 2.5 CaCl_2_, 1.3 MgSO_4_, and 10 D-glucose) at 32 °C and were continuously oxygenated with 95% O_2_ and 5% CO_2_. This was followed by 1-h incubation in oxygenated aCSF at 34 °C prior to recording. During all recordings, the slices were continuously perfused with oxygenated aCSF via a gravity-fed system. Whole-cell patch-clamp recordings were conducted from the somata of CA1 pyramidal neurons, which were voltage-clamped at a holding potential of −80 mV. Neuronal excitability was assessed by applying a 1,000-ms depolarizing current injection to evoke action potential firing. The measured parameters included spike half-width, the number of action potentials elicited, and interspike intervals. Prior to the application of BmK AS, the slices were pretreated with 5 μM kA for 10 min.

### 4.15 Data analysis

The raw data were analyzed by Origin 8.5 (OriginLab, United States). The results are shown as means ± SEM, with the number of experiments shown as n in the figure legends. Differences between means were analyzed by Student’s test or one-way ANOVA, with *P* < 0.05 showing a significant difference. For electrophysiological data normalized to each cell’s own pre-toxin control, statistical comparisons against the baseline (value of 1) were performed using a paired Student’s t-test. The voltage dependence of activation and inactivation was described by fitting the conductance-voltage or steady-state inactivation data with a Boltzmann function. Crucially, this Boltzmann fit was performed individually on the dataset from each cell to obtain cell-specific parameters (e.g., V_1_/_2_, *k*). To assess the effect of BmK AS, the parameters from the same set of cells under control conditions and after toxin perfusion were compared using a paired Student’s t-test. The averaged curves presented in the figures (e.g., Supplementary Figure S1) are Boltzmann fits to the grand mean data points at each voltage and are displayed for illustrative purposes. The reported P-values always refer to the statistical comparison of the individually fitted parameters across cells.

## Data Availability

The original contributions presented in the study are included in the article/Supplementary Material; further inquiries can be directed to the corresponding authors.
