# Supplementary material for: The underlying mechanism of scorpion venom peptide BmK AS in reducing epilepsy seizures: mediated through dual modulation of Nav1.6 and the inflammasome pathway
Source: Front Pharmacol. 2026 Apr 15;17:1747856. doi: 10.3389/fphar.2026.1747856 (PMC13125104; doi:10.3389/fphar.2026.1747856)
Supplement: Supplementary file 1 [file DataSheet1.docx]

**Supplementary Information**

Anti-epileptic Mechanism of Scorpion Extract BmK AS by Modulating Voltage-gated Sodium Channel Nav1.6.


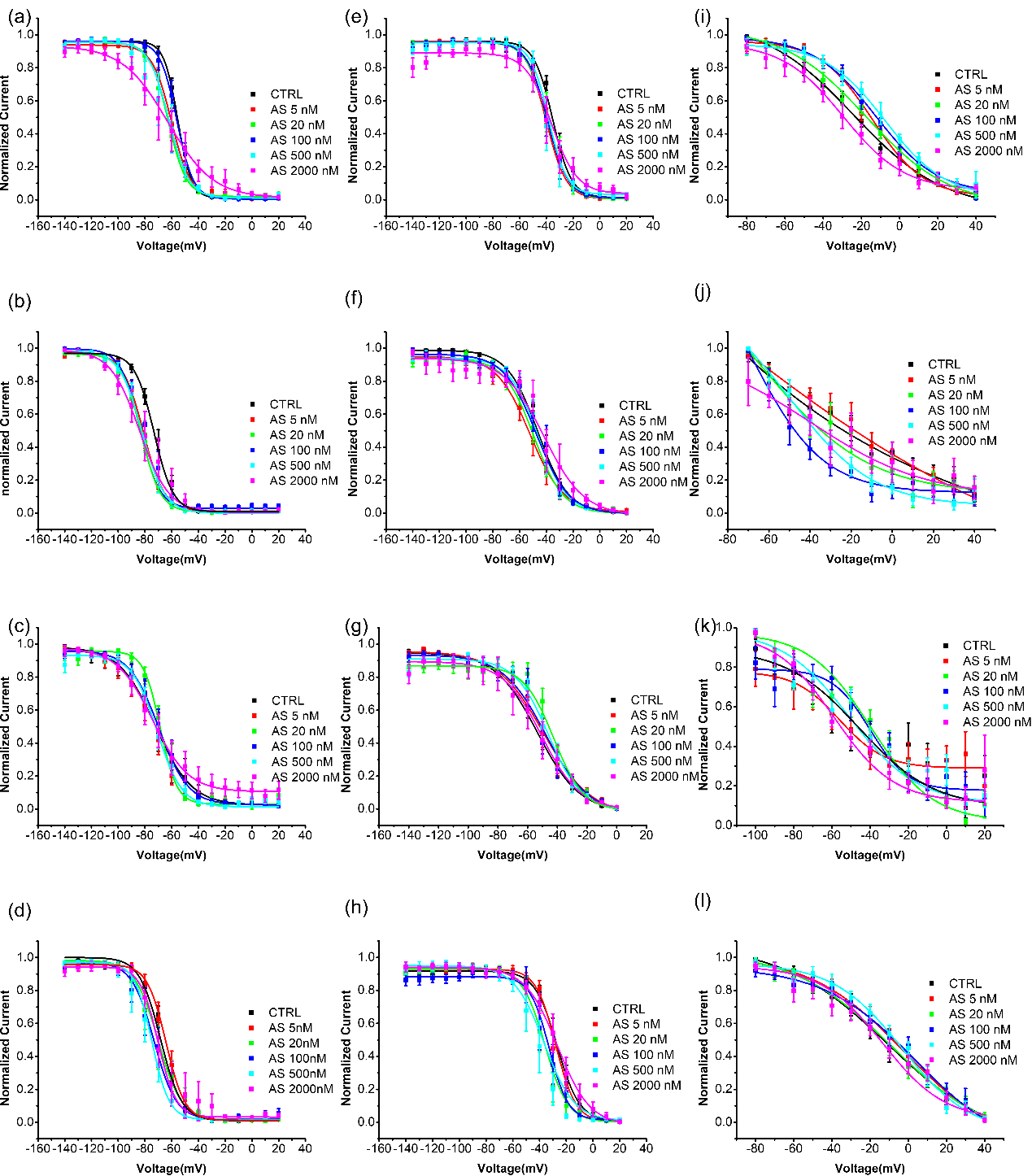


Supplementary Figure 1. Effects of BmK AS on the voltage dependence of inactivation and recovery from inactivation of VGSCs in HEK293t cells

(a-d) Steady-state inactivation for rNav1.4(a), rNav1.5(b), mNav1.6(c), and hNav1.7(d) expressed in HEK293T cells, (e-h) Fast inactivation for rNav1.4(e), rNav1.5(f), mNav1.6(g), and hNav1.7(h) expressed in HEK293T cells, and (i-1) slow inactivation curves for rNav1.4(i), rNav1.5(j), mNav1.6(k), and hNav1.7(l) expressed in HEK293T cells. Data points represent mean ± SEM of normalized current from n≥6 independent cells (see Tables S1-S4 for n≥6). Curves are Boltzmann fits to the mean data. Statistical significance for shifts in half-inactivation voltage (V_1/2_) was determined by paired Student’s t-tests comparing values obtained from individual Boltzmann fits per cell before and after BmK AS application; the detailed parameters (V_1/2_, k, and P values) are provided in Tables S1–S4.


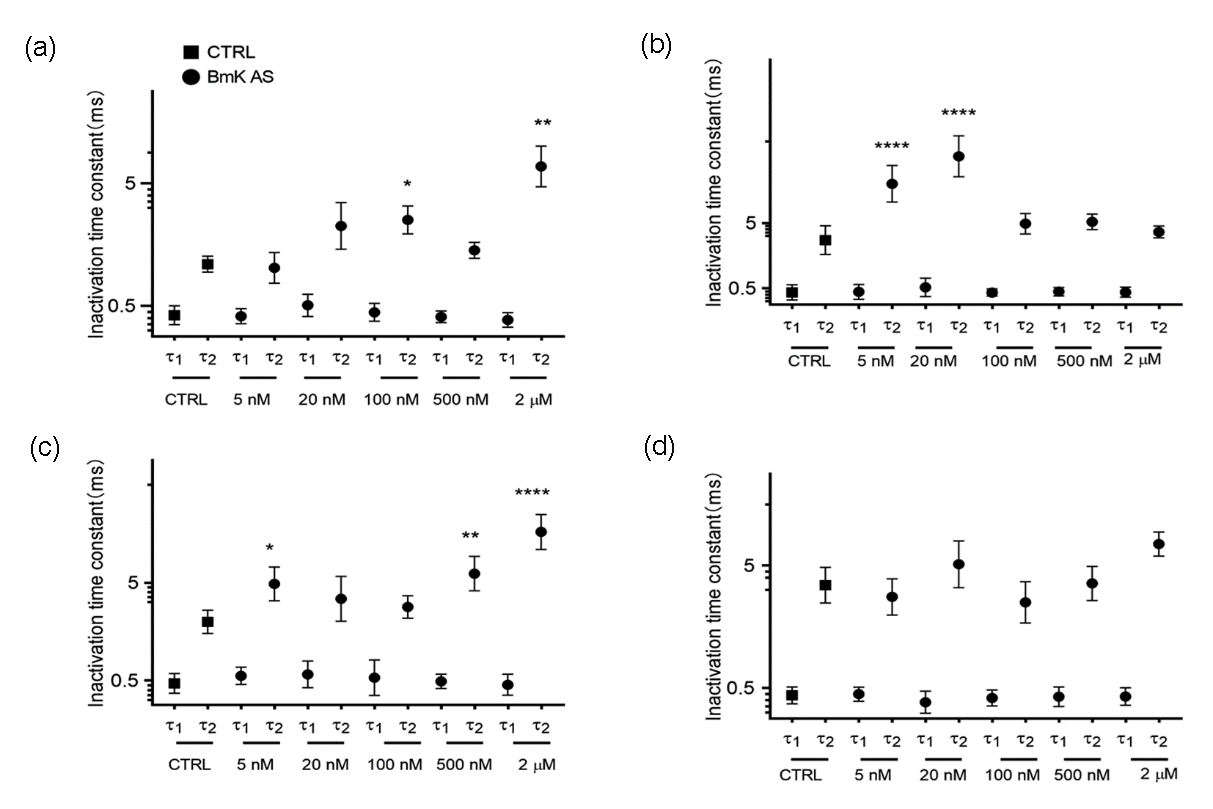


Supplementary Figure 2. Kinetic analysis of the inactivation of HEK 293T VGSCs at -10 mV in the absence and presence of BmK AS

(a-d) Fast (τ_1_, left bars) and slow (τ_2_, right bars) inactivation time constants of sodium currents at –10 mV for rNav1.4 (a), rNav1.5 (b), mNav1.6 (c), and hNav1.7 (d). Bars represent mean ± SEM. Time constants were derived from double-exponential fits to the current decay of each individual cell. Significance (*P < 0.05, **P < 0.01, ***P < 0.001) versus Control was assessed by paired Student’s t-tests on these fitted τ values.


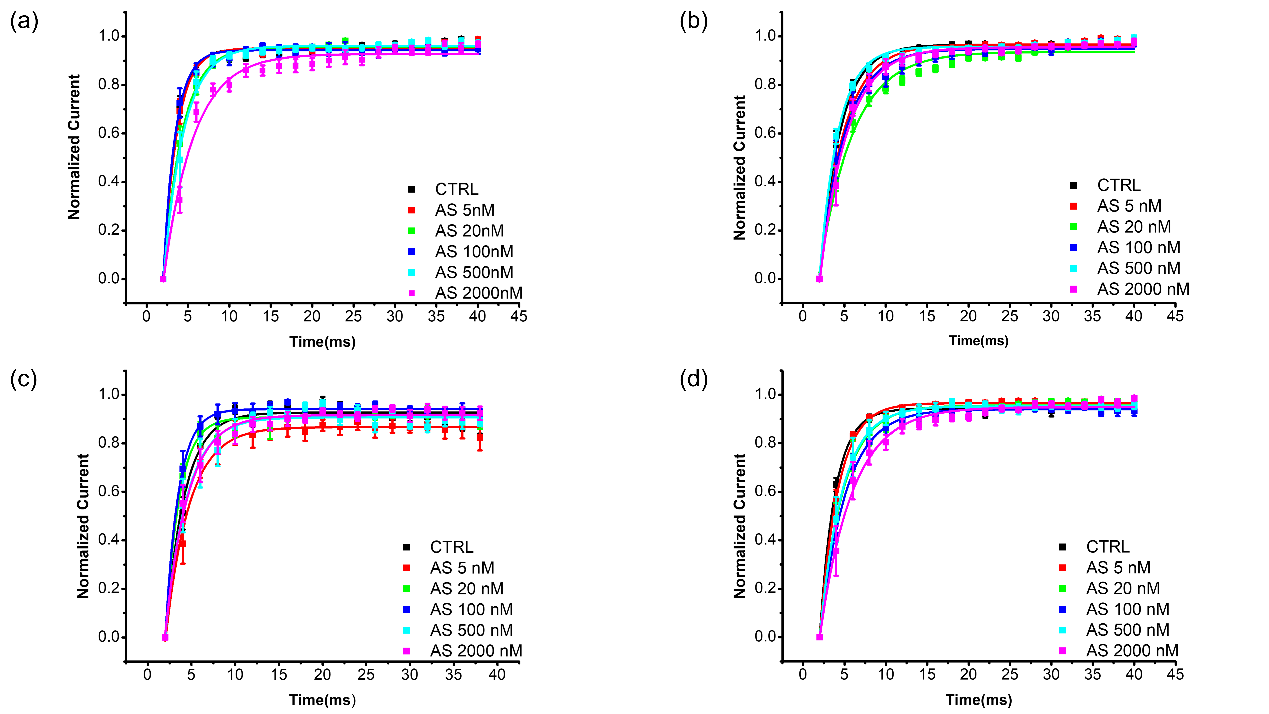


Supplementary Figure 3. Effect of BmK AS on the recovery from inactivation in HEK293t cells

(a-d) Shifts in the voltage-dependence of recovery from inactivation in the rNav1.4 after treatment with 5, 20, 100, 500 and 2000 nM BmK AS.Recovery from inactivation for rNav1.4 (a), rNav1.5 (b), mNav1.6 (c), and hNav1.7 (d). Data points show mean ± SEM of the fraction of current recovered. Curves are single-exponential fits. The recovery time constant (τ_rec) for each cell was obtained from individual exponential fits. Statistical analysis of τ_rec values (control vs. BmK AS) is presented in Tables S1–S4.


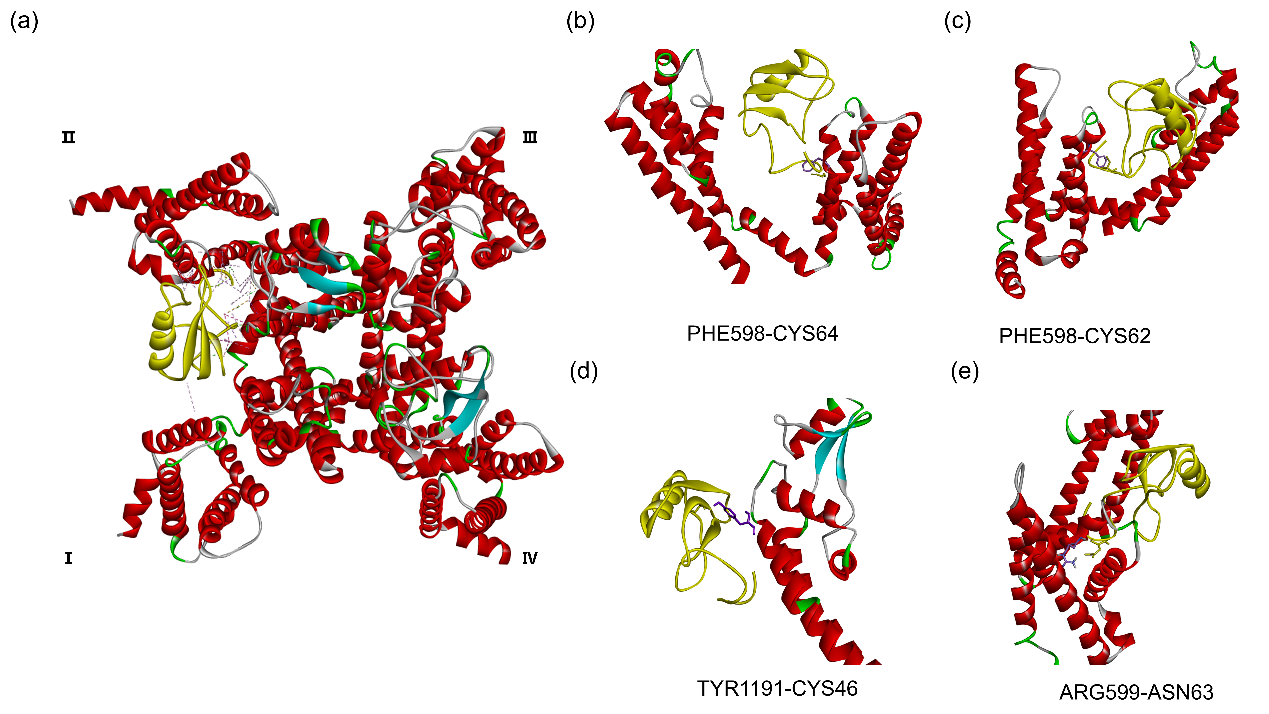


Supplementary Figure 4. Putative binding sites of BmK AS on Nav1.6 identified by molecular docking simulations. (a) The connection diagram of Nav1.6 channel and BmK AS; (b-h) Key sites for the specific interaction of BmK AS with Nav1.6 channel. The specific residues and interaction distances are listed in Table S5.

Table S1. Parameters for voltage dependence of activation, inactivation and recovery of rNav1.4.

| Concentration | CTRL | 5 nM | 20 nM | 100 nM | 500 nM | 2000 nM |
| --- | --- | --- | --- | --- | --- | --- |
| n | 13 | 8 | 8 | 8 | 8 | 8 |
| Activation |  |  |  |  |  |  |
| V_1/2_ (mV) | -26.3±0.9 | -29.1±0.9 | -36.3±0.8* | -30.5±1.1 | -32.2±1.4* | -34.0±0.9* |
| k (mV) | 7.2±0.3 | 6.4±0.3 | 5.5±0.4 | 6.0±0.4 | 6.5±0.5 | 6.4±0. |
| Fast inactivation |  |  |  |  |  |  |
| V_1/2_ (mV) | -35.2±1.0 | -40.2±1.0 | -39.2±0.9 | -39.1±1.1 | -38.3±2.1 | -36.2±2.4 |
| k (mV) | 6.9±0.6 | 7.5±0.5 | 7.9±0.3 | 7.6±0.4 | 6.7±1.1 | 9.64±1.5 |
| Slow inactivation |  |  |  |  |  |  |
| V_1/2_(mV) | -22.3±1.3 | -15.8±1.0 | -16.9±0.9 | -11.8±0.7* | -10.2±0.8** | -28.6±1.6 |
| k (mV) | 17.3±0.7 | 15.9±0. 7 | 18.7±0. 7 | 17.9±0.5 | 18.5±0.7 | 21.3±1.2 |
| Steady-state inactivation |  |  |  |  |  |  |
| V_1/2_ (mV) | -57.5±2.2 | -60.1±1.0 | -62.8±1.3 | -56.2±0.9 | -62.0±3.4 | -62.2±2.0 |
| k (mV) | 6.5±0.9 | 7.2±0.5 | 6.8±0.7 | 6.2±0.3 | 7.4±1.4 | 13.5±1.1 |
| Recovery |  |  |  |  |  |  |
| τ_rec_ (ms) | 1.5±0.1 | 1.6±0.1 | 2.3±0.1 | 1.5±0.1 | 2.4±0.1 | 3.6±0.3** |

V_1/2_, voltage midpoint activation or inactivation; K, slope factor; n, number of cells; τ, time constant. Values represent the mean ± SEM. **P* < 0.05, ***P* < 0.01, ****P* < 0.001, paired Student’s t-test. Parameters were obtained from Boltzmann or exponential fits to data from individual cells. P-values result from paired t-tests comparing the parameter sets (e.g., all control V_1/2_ values vs. all BmK AS-treated V_1/2_ values) from the same population of cells.

Table S2. Parameters for voltage dependence of activation, inactivation and recovery of rNav1.5.

| Concentration | *CTRL* | *5 nM* | *20 nM* | *100 nM* | *500 nM* | *2000 nM* |
| --- | --- | --- | --- | --- | --- | --- |
| n | 25 | 11 | 8 | 8 | 6 | 8 |
| Activation |  |  |  |  |  |  |
| V_1/2_ (mV) | -41.2±0.5 | -55.8±0.7*** | -45.6±0.7 | -51.4±0.8* | -50.1±0.3* | -39.2±1.3 |
| k (mV) | 7.7±0.3 | 10.8±0.6* | 6.5±0.4 | 4.8±0.6 | 5.3±0.1 | 6.6±0.5 |
| Fast inactivation |  |  |  |  |  |  |
| V_1/2_ (mV) | -46.4±1.0 | -52.2±1.3* | -49.4±1.8 | -47.5±1.5 | -46.4±1.5 | -41.6±2.2 |
| k (mV) | 11.4±0.5 | 12.4±0.7 | 11.0±0.9 | 12.1±0.8 | 10.7±0.7 | 14.9±1.3 |
| Slow inactivation |  |  |  |  |  |  |
| V_1/2_ (mV) | -22.5±3.8 | -17.4±2.8 | -30.2±4.3 | -37.1±3.3* | -35.4±2.0* | -27.0±2.7 |
| k (mV) | 19.9±2.9 | 18.1±1.7 | 12.5±2.2 | 11.0±1.9 | 11.9±0.7 | 19.6±2.4 |
| Steady-state inactivation |  |  |  |  |  |  |
| V_1/2_ (mV) | -75.4±1.5 | -81.2±1.0* | -82.9±1.2** | -82.2±2.3 | -82.1±2.0* | -80.9±2.5 |
| k (mV) | 9.1±0.6 | 7.8±0.4 | 7.3±0.5 | 7.2±1.1 | 8.4±0.8 | 10.9±0.6 |
| Recovery |  |  |  |  |  |  |
| τ_rec_ (ms) | 2.5±0.1 | 2.9±0.1 | 4.0±0.3* | 3.0±0.2 | 2.3±0.1 | 3.2±0.1 |

V_1/2_, voltage midpoint activation or inactivation; K, slope factor; n, number of cells; τ, time constant. Values represent the mean ± SEM. **P* < 0.05, ***P* < 0.01, ****P* < 0.001, paired Student’s t-test. Parameters were obtained from Boltzmann or exponential fits to data from individual cells. P-values result from paired t-tests comparing the parameter sets (e.g., all control V_1/2_ values vs. all BmK AS-treated V_1/2_ values) from the same population of cells.

Table S3. Parameters for voltage dependence of activation, inactivation and recovery of mNav1.6.

| Concentration | CTRL | 5 nM | 20 nM | 100 nM | 500 nM | 2000 nM |
| --- | --- | --- | --- | --- | --- | --- |
| n | 15 | 7 | 6 | 7 | 8 | 8 |
| Activation |  |  |  |  |  |  |
| V_1/2_ (mV) | -41.6±0.5 | -44.0±0.9 | -47.8±1.0* | -52.1±0.4*** | -43.5±0.7 | -36.7±0.9 |
| k (mV) | 7.3±0.2 | 5.5±0.3 | 2.1±0.3* | 2.6±0.2** | 4.6±0.3 | 6.1±0.4 |
| Fast inactivation |  |  |  |  |  |  |
| V_1/2_ (mV) | -53.3±1.3 | -49.1±1.8 | -42.4±1.4* | -47.8±1.9 | -46.0±1.9* | -50.5±2.4 |
| k (mV) | 14.2±0.8 | 15.7±1.3 | 10.0±1.1** | 14.3±1.4 | 11.1±1.2* | 12.9±1.6 |
| Slow inactivation |  |  |  |  |  |  |
| V_1/2_(mV) | -45.6±2.8 | -39.6±3.2 | -41.4±2.4 | -40.0±2.8 | -50.8±4.6 | -58.7±1.5* |
| k (mV) | 19.4±2.1 | 16.1±2.1 | 17.0±2.2 | 9.8±2.2 | 17.7±2.9 | 15.6±1.6 |
| Steady-state inactivation |  |  |  |  |  |  |
| V_1/2_(mV) | -72.1±1.4 | -77.1±1.3 | -68.6±1.1 | -69.6±0.7 | -70.9±1.7 | -74.2±1.1 |
| k (mV) | 13.5±0.9 | 11.1±0.8 | 6.2±0.6 | 11.0±0.4 | 9.2±1.0 | 13.4±0.8 |
| Recovery |  |  |  |  |  |  |
| τ_rec_ (ms) | 2.2±0.2 | 2.7±0.2 | 1.6±0.1 | 1.5±0.1 | 2.4±0.2 | 2.5±0.1 |

V_1/2_, voltage midpoint activation or inactivation; K, slope factor; n, number of cells; τ, time constant. Values represent the mean ± SEM. **P* < 0.05, ***P* < 0.01, ****P* < 0.001, paired Student’s t-test. Parameters were obtained from Boltzmann or exponential fits to data from individual cells. P-values result from paired t-tests comparing the parameter sets (e.g., all control V_1/2_ values vs. all BmK AS-treated V_1/2_ values) from the same population of cells.

Table S4. Parameters for voltage dependence of activation, inactivation and recovery of hNav1.7.

| Concentration | CTRL | 5 nM | 20 nM | 100 nM | 500 nM | 2000 nM |
| --- | --- | --- | --- | --- | --- | --- |
| n | 14 | 8 | 8 | 6 | 7 | 7 |
| Activation |  |  |  |  |  |  |
| V_1/2_ (mV) | -28.8±1.7 | -39.3±1.1** | -37.7±0.8** | -34.3±0.9 | -36.2±0.9* | -36.5±0.9* |
| k (mV) | 4.4±0.5 | 4.3±0.3 | 3.3±0.4 | 4.3±0.3 | 4.5±0.3 | 6.2±0.3* |
| Fast inactivation |  |  |  |  |  |  |
| V_1/2_(mV) | -26.7±0.5 | -27.8±0.9 | -35.8±1.3* | -32.7±1.8 | -36.5±1.9* | -26.5±2.0 |
| k (mV) | 8.3±0.3 | 7.4±0.5 | 8.5±0.7 | 7.1±0.8 | 11.3±1.1 | 12.1±0.6 |
| Slow inactivation |  |  |  |  |  |  |
| V_1/2_ (mV) | -10.9±3.7 | -11.1±1.7 | -13.6±1.2 | -13.0±2.4 | -9.72±3.2 | -13.4±2.4 |
| k (mV) | 27.5±4.0 | 19.1±1.1 | 20.4±0.8 | 18.3±1.7 | 25.2±3.1 | 16.2±1.6 |
| Steady-state inactivation |  |  |  |  |  |  |
| V_1/2_ (mV) | -68.3±1.5 | -64.2±1.3 | -70.1±1.7 | -73.2±1.8 | -74.9±2.8 | -70.1±1.7 |
| k (mV) | 7.9±0.9 | 7.0±0.6 | 8.8±0.6 | 8.3±0.8 | 6.5±1.2 | 6.7±1.2 |
| Recovery |  |  |  |  |  |  |
| τ_rec_ (ms) | 1.8±0.1 | 2.2±0.1 | 2.8±0.1 | 3.1±0.1 | 2.7±0.1 | 3.9±0.2** |

V_1/2_, voltage midpoint activation or inactivation; K, slope factor; n, number of cells; τ, time constant. Values represent the mean ± SEM. **P* < 0.05, ***P* < 0.01, ****P* < 0.001, paired Student’s t-test. Parameters were obtained from Boltzmann or exponential fits to data from individual cells. P-values result from paired t-tests comparing the parameter sets (e.g., all control V_1/2_ values vs. all BmK AS-treated V_1/2_ values) from the same population of cells.

Table S5. The distance between ReceptorResidue and LigandResidue obtained in the docking simulation of BmK AS with Nav1.6

| ReceptorResidue | LigandResidue | Distance | Type |
| --- | --- | --- | --- |
| 3:TYR1191 | Ligand:CYS46 | 1.8607 | Hydrogen Bonds |
| 2:ARG599 | Ligand:ASN63 | 2.0387 | Hydrogen Bonds |
| 3:SER1177 | Ligand:TYR38 | 2.0510 | Hydrogen Bonds |
| 2:PHE598 | Ligand:GLY64 | 2.4767 | Pi-Lone Pair |
| 2:PHE598 | Ligand:CYS62 | 2.7923 | Pi-Lone Pair |
| 3:TYR1191 | Ligand:ASN2 | 3.4654 | Pi-Amide Stacked |
| 3:ILE1198 | Ligand:TYR57 | 3.5483 | Pi-Sigma |
| 3:TYR1191 | Ligand:GLY3 | 3.5796 | Pi-Amide Stacked |
| 3:LYS1179 | Ligand:PHE39 | 3.6141 | Pi- Sigma |
